# Dietary sulforaphane in cancer chemoprevention: epigenetic regulation and PRMT5-MEP50 complex inhibition-a systematic review

**DOI:** 10.3389/fphar.2026.1865620

**Published:** 2026-08-04

**Authors:** Ping Yan, Changfeng Sun, Cunliang Deng

**Affiliations:** 1 Department of Infectious Diseases, The Affiliated Hospital, Southwest Medical University, Luzhou, China; 2 Laboratory of Infection and Immunity, The Affiliated Hospital, Southwest Medical University, Luzhou, China; 3 Department of Gastroenterology, The People’s Hospital of Gulin County, Luzhou, China

**Keywords:** cancer chemoprevention, epigenetics, MEP50, precision prevention, PRMT5, sulforaphane, systematic review

## Abstract

**Background:**

Cancer chemoprevention using dietary bioactive compounds has emerged as a promising, cost-effective strategy to reduce global cancer incidence and mortality. Sulforaphane (SFN), an isothiocyanate abundant in cruciferous vegetables, has attracted considerable attention for its pleiotropic anticancer effects.

**Methods:**

This systematic review synthesizes evidence through February 2026, with a particular focus on SFN’s roles in epigenetic remodeling and modulating the PRMT5-MEP50 complex. Following PRISMA 2020 guidelines and a PROSPERO-registered protocol (Registration ID: CRD420261435174), we identified 118 eligible studies (17 clinical trials, 31 observational studies, and 70 preclinical mechanistic investigations). Study quality was assessed using Cochrane RoB 2.0, the Newcastle-Ottawa Scale, and SYRCLE’s risk-of-bias tool.

**Results:**

Recent clinical evidence includes a randomized Phase II trial in high-risk former smokers, in which 12-month supplementation with SFN (95 μmol/day) reduced the bronchial Ki-67 proliferation index by 20%. Meanwhile, the placebo group showed a 65% increase. Across clinical studies, SFN and SFN-rich preparations were well tolerated and associated with favorable changes in proliferation markers, histone acetylation, and tumor suppressor gene expression. Mechanistically, SFN modulates epigenetic networks by inhibiting DNA methyltransferases and histone deacetylases, activating TET-mediated DNA demethylation, and regulating microRNAs and long non-coding RNAs. In parallel, converging preclinical data indicate that SFN targets the oncogenic PRMT5-MEP50 complex through four complementary mechanisms: (1) proteasome-dependent degradation of PRMT5 and MEP50; (2) reduction of PRMT5-MEP50 complex formation; (3) inhibition of histone and non-histone arginine methylation activity; and (4) modulation of PRMT5 subcellular localization, with selective attenuation of cytoplasmic oncogenic functions. Computational molecular docking and cellular co-immunoprecipitation (Co-IP) support the hypothesis that SFN interferes with PRMT5-MEP50 protein-protein interactions (PPIs). Nevertheless, label-free biophysical assays (surface plasmon resonance (SPR), isothermal titration calorimetry (ITC), nuclear magnetic resonance (NMR)) using purified holocomplexes are still lacking to validate direct high-affinity binding between SFN and PRMT5-MEP50. Together, these actions suggest an “epigenetic-PRMT5 inhibition” dual-axis framework that cooperatively reactivates silenced tumor suppressor programs and restricts oncogenic signaling.

**Conclusion:**

We discuss key translational issues, including bioavailability limitations, inter-individual variability driven by GST genotypes and gut microbiota, and design considerations for genotype-guided Phase III chemoprevention trials. SFN appears to exemplify a dietary phytochemical with mechanism-based selectivity for cancer cells at nutritional concentrations, an excellent safety profile, and emerging biomarker evidence from randomized trials. If ongoing formulation and precision prevention strategies validate current findings in large-scale trials, SFN may inform the development of safe, accessible, and mechanism-guided cancer chemopreventive interventions.

**Systematic Review Registration:**

https://www.crd.york.ac.uk/prospero/, identifier CRD420261435174.

## 1 Introduction

Cancer remains a leading cause of morbidity and mortality worldwide. According to the latest GLOBOCAN 2022 statistics published in 2024, there were approximately 20.0 million new cancer cases, and 9.7 million cancer deaths globally in 2022, and the annual cancer incidence is projected to rise to 35 million new cases by 2050 under current trends (Bray et al., 2024; Filho et al., 2025; Son et al., 2026). Tumorigenesis is a complex, multistep process driven by the accumulation of genetic and epigenetic alterations that disrupt core cellular processes, including uncontrolled proliferation, evasion of apoptosis, impaired DNA repair, and metastatic dissemination (Hanahan and Weinberg, 2011). In contrast to irreversible genetic mutations, epigenetic modifications-heritable changes in gene expression without alterations in DNA sequence-are dynamic and potentially reversible, making them attractive targets for cancer chemoprevention (Baylin and Jones, 2011).

Dietary bioactive compounds represent a particularly promising class of epigenetic modulators, offering safe, widely accessible, and cost-effective interventions with generally favorable safety profiles compared with synthetic chemopreventive agents (Surh, 2003). Sulforaphane (SFN, 1-isothiocyanato-4-methylsulfinylbutane) is a well-characterized secondary metabolite generated from the hydrolysis of glucoraphanin, a glucosinolate enriched in cruciferous vegetables, by the enzyme myrosinase (Fahey et al., 1997). Initially identified in the 1990s as a potent inducer of phase II detoxification enzymes (Fahey et al., 2002), SFN has since been extensively investigated for its broad-spectrum anticancer activities.

Recent work has highlighted SFN’s capacity to modulate epigenetic networks and interfere with the oncogenic protein arginine methyltransferase 5 (PRMT5) complex (Surh, 2003; Recillas-Targa, 2022; Dashwood and Ho, 2007). PRMT5 is the major type II protein arginine methyltransferase that, together with its obligate co-factor MEP50 (WDR77), forms a hetero-octameric complex catalyzing symmetric dimethylation of both histone and non-histone substrates (Stopa et al., 2015; Wu et al., 2021). PRMT5 is frequently overexpressed in diverse malignancies, where it contributes to tumor suppressor gene silencing, activation of oncogenic signaling pathways, maintenance of cancer stem cells, therapy resistance, and immune evasion (Li et al., 2021; Gómez de Cedrón et al., 2023).

Between 2023 and 2025, multiple synthetic PRMT5 inhibitors advanced into early-phase clinical trials (Sabnis, 2021; Feustel and Falchook, 2022; Shah and Rodon, 2025). Although these agents demonstrate on-target activity, their broad catalytic inhibition and off-target toxicity have limited their therapeutic window in some settings, as shown in Table 1. In parallel, SFN has emerged as a prototypical natural modulator of the PRMT5-MEP50 complex, acting with selectivity against cancer cells at concentrations achievable through dietary intake or supplementation. To our knowledge, SFN is among the first dietary phytochemicals reported to perturb PRMT5-MEP50-dependent signaling, with molecular docking and cellular co-immunoprecipitation (Co-IP) data suggesting possible interference with PRMT5-MEP50 protein-protein interactions, although direct high-affinity binding remains to be demonstrated.

**TABLE 1:** Clinical-stage synthetic PRMT5 inhibitors.

Inhibitor	Mechanism	Clinical phase	Main cancer types	Current status & key outcomes	Key limitations	Sample size (N)	Clinical endpoints	Statistical significance
GSK3326595	SAM-competitive catalytic inhibitor	Phase II	Solid tumors, lymphoma	On-target activity; limited by myelosuppression	Broad inhibition, hematologic toxicity	≥100	Objective response rate (ORR); progression-free survival (PFS); target engagement	Not reported; descriptive efficacy signals
JNJ-64619178	Catalytic inhibitor	Phase II	Advanced solid tumors	Preliminary target engagement; dose interruptions	Fatigue, thrombocytopenia	≥80	ORR,PFS, safety profile	Not reported; dose-limiting toxicity observed
PRT543	Selective catalytic inhibitor	Phase Ib	Myeloid malignancies	Clinical activity in splicing-mutant tumors	Gastrointestinal and hematologic toxicity	≥50	ORR; duration of response (DOR); safety	Descriptive; no formal statistical testing
BMS-986504	MTA-cooperative (MTAP-deleted)	Phase II/III	NSCLC, pancreatic cancer	Synthetic lethality in MTAP-del; well-tolerated	Restricted to MTAP-deleted patients	≥200	Overall survival (OS); PFS; ORR	Under statistical analysis; interim data favorable
AMG 193	MTA-cooperative	Phase I	MTAP-deleted solid tumors	Confirmed target engagement; acceptable safety	Early stage; limited efficacy data	≥30	Maximum tolerated dose (MTD); target engagement	Descriptive; no formal significance testing
BGB-58067	MTA-cooperative, brain-penetrant	Phase I/II	MTAP-del solid tumors, glioma	Preclinical CNS activity; ongoing FIH	Early development	≥40	MTD; CNS target engagement; safety	Descriptive; ongoing enrollment
CS08399	MTA-cooperative	Phase I	MTAP-del tumors	IND-approved; first-in-human ongoing	Early stage	≥20	MTD; safety; pharmacokinetics (PK)	Descriptive; no formal significance testing

This systematic review, conducted in accordance with PRISMA 2020 guidelines, integrates epidemiologic, clinical, and mechanistic evidence on SFN with an emphasis on the interplay between epigenetic regulation and PRMT5-MEP50 modulation. While our review follows a structured, protocol-driven search, the synthesis is primarily narrative and mechanism-focused due to substantial heterogeneity in study designs, interventions, and outcomes. We also evaluate the translational potential of SFN as a component of precision chemoprevention strategies.

## 2 Methodology

### 2.1 Literature search strategy and registration

This systematic review was prospectively registered with PROSPERO before literature retrieval (Registration ID: CRD420261435174). We searched PubMed, Embase, Web of Science, the Cochrane Library, and Google Scholar from database inception to 15 February 2026. The search strategy was informed by the PICOS framework: Population (individuals with cancer or at increased cancer risk, as well as relevant preclinical cancer models), Intervention (sulforaphane or SFN-rich cruciferous vegetable intake), Comparison (placebo, standard care, or no intervention), Outcomes (cancer incidence, premalignant lesions, tumor burden, proliferation and apoptosis markers, epigenetic modifications, PRMT5/MEP50 expression and activity), and Study design (randomized controlled trials, cohort and case-control studies, and mechanistic preclinical studies).

The PubMed strategy combined Medical Subject Headings (MeSH) and free-text terms for sulforaphane and related isothiocyanates with terms for cancer, chemoprevention, epigenetics, and PRMT5-related pathways. Analogous strategies were adapted for other databases. Google Scholar was used to identify additional gray literature and recent articles not yet indexed, limited to the first 200 hits per query. We manually screened reference lists of key reviews and primary studies and contacted domain experts to identify additional or unpublished data. The full electronic search strategies for all databases are available from the corresponding author upon reasonable request.

### 2.2 Inclusion and exclusion criteria

Studies were eligible if they: (1) investigated SFN or SFN-rich cruciferous vegetables in the context of cancer prevention, early intervention, or treatment; (2) reported outcomes related to epigenetic mechanisms (DNA methylation, histone modifications, noncoding RNAs) and/or PRMT5-related pathways; (3) used designs including randomized or non-randomized clinical trials, prospective cohort studies, case-control studies, or mechanistic *in vitro*/*in vivo* preclinical studies; (4) were published in English; and (5) provided sufficient data for extraction.

Exclusion criteria were: (1) non-English publications; (2) publications restricted to abstracts, conference proceedings without full data, editorials, or narrative reviews; (3) studies where SFN was used only in complex mixtures and independent effects could not be distinguished; (4) case reports and case series without comparators; and (5) duplicate data from overlapping study populations (in which case the most complete or recent report was retained).

Although our primary focus was chemoprevention, we also included early-phase therapeutic and mechanistic studies that provided critical insights into SFN’s epigenetic and PRMT5-related mechanisms relevant to tumor initiation, progression, or recurrence.

### 2.3 Study screening and data extraction

Two reviewers independently screened titles and abstracts using Covidence software. Full-text articles were retrieved for potentially eligible records. Discrepancies were resolved by discussion or by consultation with a third reviewer.

From each included study, we extracted: (1) bibliographic information (first author, year, country); (2) study design and setting; (3) participant or model characteristics (cancer or precancerous condition, sample size, cell lines or animal models); (4) intervention details (SFN dose or concentration, formulation, route and duration of administration, comparator where applicable); (5) outcomes (cancer incidence or recurrence, tumor burden, proliferation and apoptosis markers, epigenetic modifications, PRMT5/MEP50 expression and downstream markers such as H4R3me2s); and (6) quality indicators relevant to the study design (randomization, blinding, allocation concealment, sample size calculation, adjustment for confounders).

### 2.4 Quality assessment and risk of bias

Quality assessment tools were selected according to the study design. Randomized controlled trials (RCTs) were evaluated using the Cochrane Risk of Bias 2.0 (RoB 2.0) tool, focusing on randomization, deviations from intended interventions, missing outcome data, outcome measurement, and selective reporting. Cohort and case-control studies were assessed using the Newcastle-Ottawa Scale (NOS), with attention to selection, comparability, and ascertainment of outcome or exposure. Animal experiments were assessed using SYRCLE’s Risk of Bias tool, covering sequence generation, baseline characteristics, allocation concealment, randomization, blinding, outcome assessment, incomplete outcome data, and selective reporting.

Studies were not excluded solely on the basis of risk-of-bias assessment. However, findings from studies judged to be at high risk of bias in critical domains were interpreted cautiously. These studies are indicated with an asterisk in the corresponding summary tables and explicitly flagged in the Discussion where relevant.

### 2.5 Data synthesis strategy and heterogeneity assessment

Given the substantial, multi-dimensional heterogeneity anticipated across study designs, populations, interventions, and outcomes, we prespecified that no formal quantitative meta-analysis would be undertaken. Instead, we adopted a structured narrative synthesis approach. Before data extraction, we defined a framework to characterize and handle heterogeneity across five domains: (1) study populations and cancer subtypes; (2) intervention regimens; (3) outcome biomarkers and measurement protocols; (4) methodological risk-of-bias profiles; and (5) publication and technical biases.

Heterogeneity in study populations and cancer subtypes arose from integrating three distinct evidence tiers: clinical intervention trials in high-risk or early-stage patients, observational epidemiologic studies in general and high-risk populations, and preclinical *in vitro*/*in vivo* models encompassing multiple tumor types, each with different baseline risks and clinical contexts. Intervention regimens varied widely with SFN source (whole-food vs. extract or supplement), nominal dose, duration, and route of administration, as well as biological modifiers of exposure such as GST genotypes, myrosinase activity, and gut microbiota composition. Outcome biomarkers and measurement protocols were equally diverse: clinical and epidemiologic studies assessed heterogeneous clinical and surrogate endpoints (e.g., Ki-67 indices, adenoma recurrence, prostate-specific antigen (PSA) kinetics, site-specific cancer incidence), while mechanistic studies focused on a broad array of epigenetic and PRMT5-related readouts measured by non-standardized laboratory platforms and analytic pipelines. Methodological risk-of-bias profiles differed across randomized trials, non-randomized clinical studies, observational designs, and animal experiments with respect to randomization, blinding, confounder control, and selective reporting, and these differences were not evenly distributed across cancer sites or SFN regimens. Finally, publication and technical biases were suggested by the presence of registered but unpublished SFN chemoprevention trials and by substantial inter-laboratory variation in key molecular assays (e.g., Co-IP, ChIP, flow cytometry, methylation profiling), which could not be quantitatively harmonized.

Because these sources of heterogeneity are structural and cannot be adequately modeled within standard meta-analytic assumptions, we judged that pooling effect sizes would likely yield spuriously precise and potentially misleading summary estimates. Therefore, for each eligible study, we extracted data into standardized templates according to study type (clinical trial, observational study, or preclinical mechanistic investigation) and synthesized the evidence qualitatively within and across these tiers, focusing on consistency of direction, convergence between epidemiologic, clinical, and mechanistic findings, and biological plausibility rather than on formal pooled effect estimates.

## 3 Results

### 3.1 Study selection and overall evidence base

**FIGURE 1:**
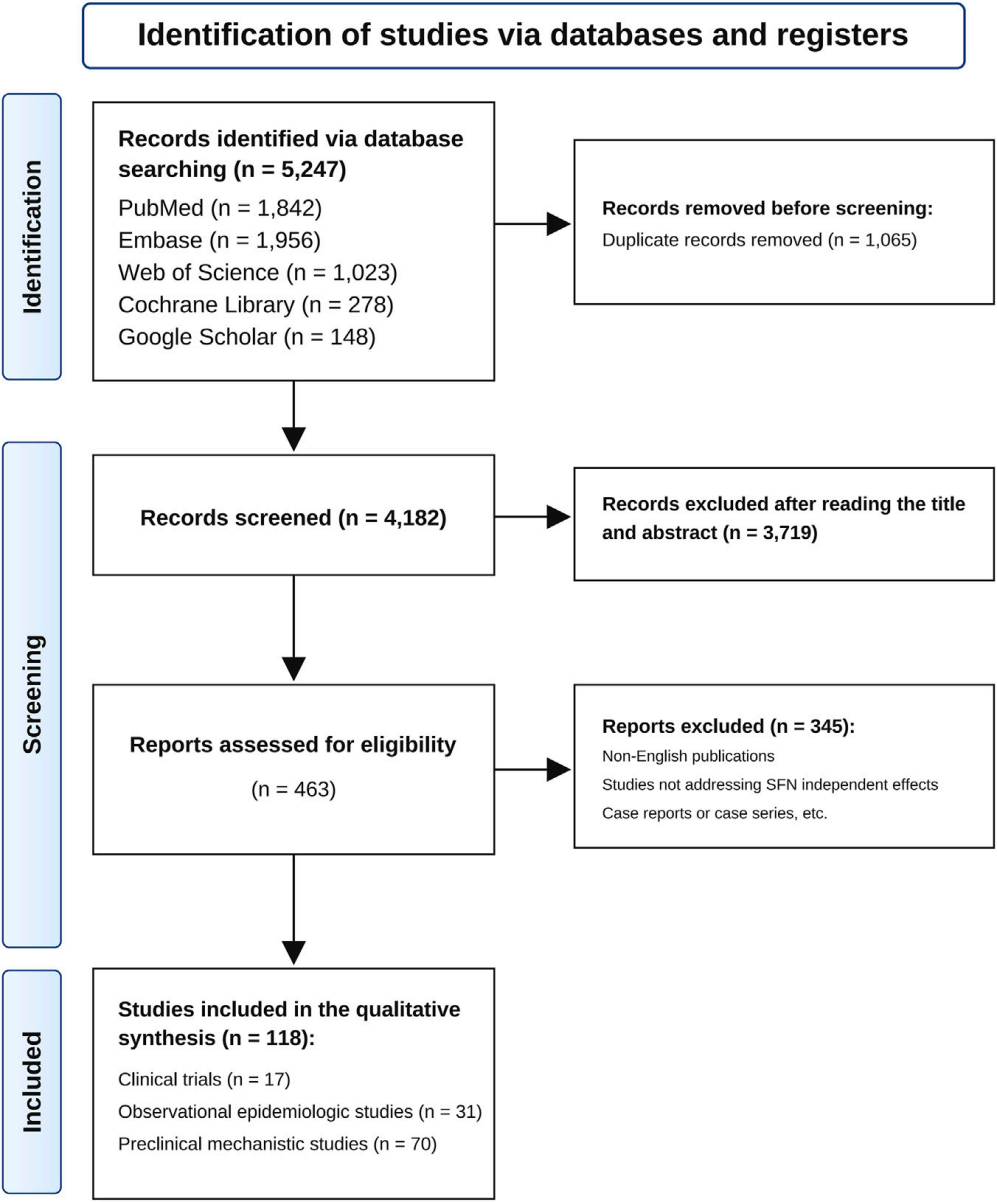
PRISMA 2020 literature screening flow diagram showing systematic identification, screening, eligibility assessment, and inclusion of 118 studies.

The electronic search and manual screening yielded 5,247 records. After removal of duplicates, 4,182 unique records remained for title and abstract screening. Of these, 463 articles were selected for full-text assessment. Ultimately, 118 studies met the inclusion criteria and were included in the qualitative synthesis (Figure 1). These comprised 17 clinical trials, 31 observational epidemiologic studies (14 prospective cohort and 17 case-control studies), and 70 preclinical mechanistic investigations (42 *in vitro* and 28 *in vivo*).

Clinical trials predominantly enrolled individuals with or at elevated risk for prostate, breast, lung, and colorectal cancers, as well as patients with recurrent respiratory papillomatosis. Observational studies focused mainly on lung, colorectal, breast, and prostate cancers in general populations or defined high-risk groups. Preclinical studies encompassed a broad spectrum of solid and hematologic malignancies, providing mechanistic support for the epidemiologic and clinical observations.

### 3.2 Study characteristics

Across the 17 clinical trials, SFN or SFN-rich preparations were administered orally at nominal doses generally ranging from 95 to 400 μmol/day for durations up to 12 months. Most trials evaluated surrogate endpoints related to proliferation, epigenetic modulation, or PRMT5-linked pathways in target tissues. SFN was generally well tolerated, with no serious treatment-related adverse events reported. SFN was administered in various formulations (e.g., broccoli sprout extract, standardized SFN-rich preparations) at doses typically ranging from 95 to 400 μmol/day for durations up to 12 months. Across these trials, SFN was generally safe and surrogate biomarker improvements included suppressed Ki-67 proliferation indices, reduced histone deacetylase (HDAC) enzymatic activity, and elevated tumor suppressor gene (TSG) expression.

Observational studies included 14 prospective cohorts and 17 case-control investigations across multiple cancer types, most commonly lung, colorectal, breast, and prostate cancers. These studies consistently reported inverse associations between high cruciferous vegetable intake and cancer risk, with relative risk reductions typically ranging from 10% to 30%, varying by cancer site and genetic background (e.g., GST and CYP polymorphisms).

Preclinical mechanistic studies employed a wide range of human cancer cell lines, including prostate, lung, breast, colorectal, mesothelioma, skin, liver, and hematologic malignancies. These experiments consistently demonstrated that SFN inhibits DNA methyltransferases (DNMTs) and HDACs, modulates histone methylation, regulates microRNAs (miRNAs) and long non-coding RNAs (lncRNAs), and interferes with PRMT5-MEP50-dependent signaling. *In vivo* animal models corroborated these findings, with SFN supplementation leading to 40%–80% reductions in tumor volume or number and minimal systemic toxicity.

Consistent with the pre-specified five-domain heterogeneity framework described in Section 2.5, the 118 included studies exhibited prominent cross-study variability across participant populations, SFN intervention regimens, outcome measurement protocols, risk-of-bias distributions, and publication status. The multidimensional heterogeneity observed in the final study pool further supported our *a priori* decision to conduct qualitative narrative synthesis rather than quantitative meta-analysis (Table 2).

**TABLE 2:** Summary of study types, cancer sites, and key findings.

Study type	Number	Primary cancer types	Key findings
Phase I/II clinical trials	17	Prostate, breast, lung, colorectal, recurrent papillomatosis	SFN safe; favorable biomarker changes (Ki-67, HDAC, p21, PTEN/p53)
Prospective cohort studies	14	Lung, colorectal, breast, prostate	High cruciferous intake associated with 15–25% risk reduction
Case-control studies	17	Lung, colorectal, breast, prostate, others	High intake associated with 10–30% risk reduction
*In vitro* mechanistic	42	Mesothelioma, skin, liver, prostate, lung, breast, colorectal	SFN inhibits DNMTs, HDACs, PRMT5; modulates miRNA/lncRNA
*In vivo* animal studies	28	Above cancer types	40–80% reduction in tumor volume/number; no major toxicity

### 3.3 Quality assessment results

#### 3.3.1 Randomized controlled trials

**FIGURE 2:**
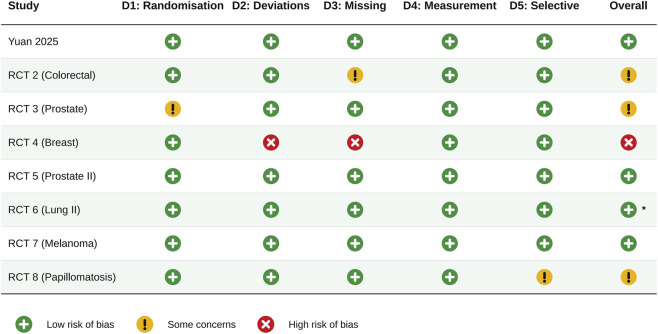
Risk-of-bias summary for the eight included randomized controlled trials assessed using the Cochrane RoB 2.0 tool. Domains assessed: D1 (randomization process), D2 (deviations from intended interventions), D3 (missing outcome data), D4 (measurement of the outcome), D5 (selection of the reported result). Green (+) indicates low risk, yellow (−) indicates some concerns, red (×) indicates high risk.

Eight RCTs were assessed with the Cochrane Risk of Bias 2.0 (RoB 2.0) tool. The remaining nine were single-arm Phase I/II studies and were evaluated separately using appropriate tools for non-randomized intervention studies (ROBINS-I) where applicable. Among the eight RCTs, the Phase II lung cancer chemoprevention trial in high-risk former smokers (Yuan et al., 2025) demonstrated low risk of bias across all five domains, with strengths including centralized computer-generated randomization, allocation concealment, and blinded outcome assessment via central pathology review (Yuan et al., 2025). Four additional RCTs were judged to be at low overall risk of bias. Two RCTs raised concerns: one colorectal adenoma trial due to approximately 15% dropout without a pre-specified intention-to-treat analysis, and one prostate cancer trial due to unclear reporting of the randomization procedures. One RCT in breast cancer prevention was at high risk of bias owing to incomplete outcome data (>20% loss to follow-up) and lack of blinding. The RoB 2.0 summary is presented as a traffic light plot (Figure 2).

#### 3.3.2 Animal experiments

Twenty-eight *in vivo* animal studies were evaluated with SYRCLE’s risk-of-bias tool. Overall methodological quality was moderate:

- Sequence generation: 19 studies (68%) explicitly described random allocation to experimental groups.
- Baseline characteristics: 22 studies (79%) provided adequate baseline comparability data.
- Allocation concealment: None of the studies (0%) reported allocation concealment procedures.
- Random housing: 2 studies (7%) described random housing of animals.
- Blinding of caregivers/investigators: 8 studies (29%) reported blinding of personnel.
- Random outcome assessment: 9 studies (32%) documented blinded outcome assessment.
- Incomplete outcome data: 21 studies (75%) were judged at low risk of attrition bias.
- Selective reporting: 16 studies (57%) were at unclear risk due to the absence of pre-registered protocols.

**FIGURE 3:**
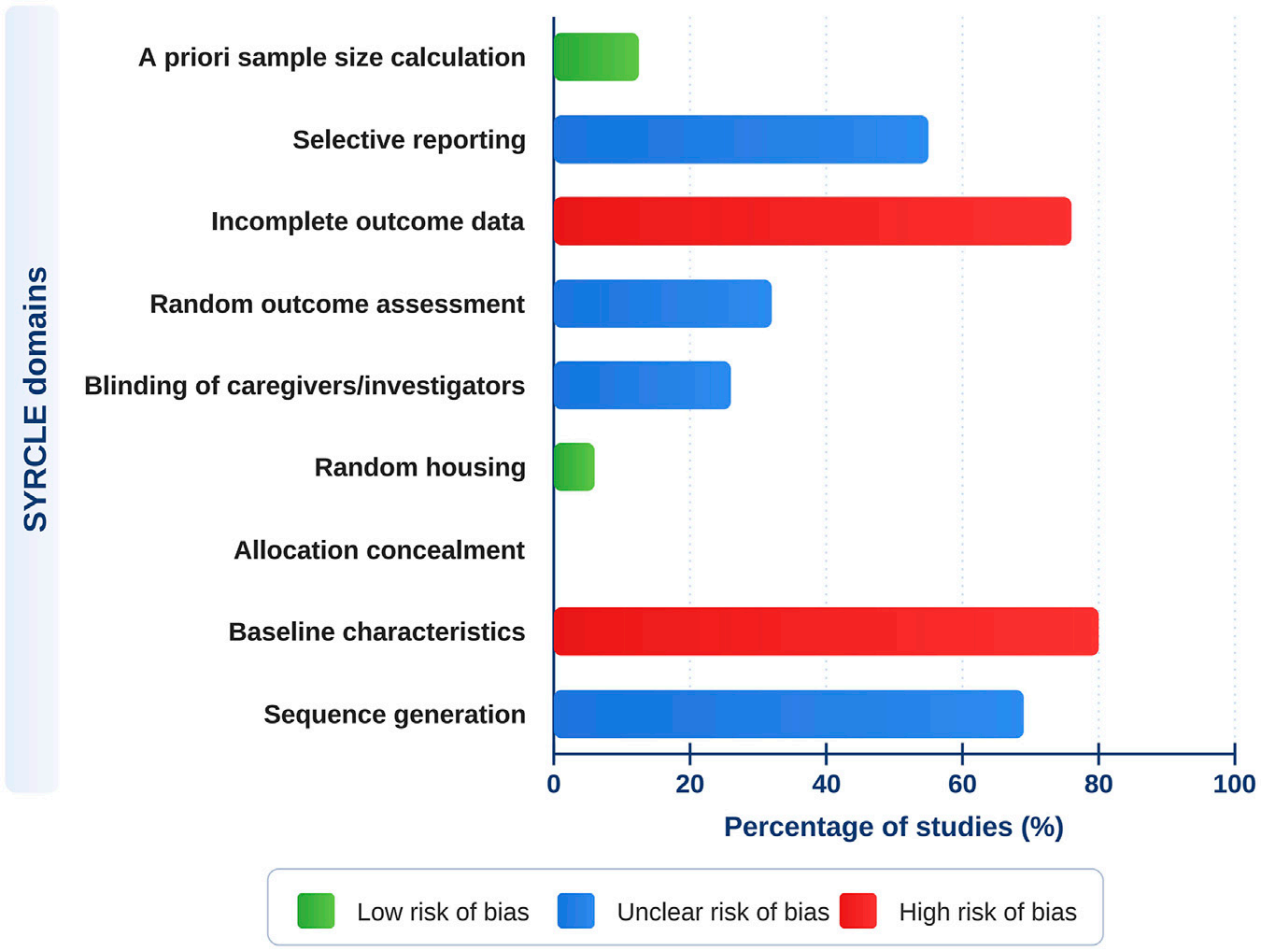
Summary of risk of bias for the 28 included animal studies, assessed using the SYRCLE tool. The bar chart shows the percentage of studies with low (green), unclear (yellow), or high (red) risk of bias in each domain.

Only 4 studies (14%) reported *a priori* sample size calculations. The most frequent potential sources of bias were inadequate allocation concealment, lack of randomization in housing allocation, and incomplete reporting of blinding procedures. A summary bar chart of risk of bias across all domains is provided (Figure 3).

#### 3.3.3 Observational studies

The 14 prospective cohort studies were assessed using the NOS. The median NOS score was 7 out of 9 (range, 5–8), indicating generally good quality. Strengths included representative cohort selection and adequate follow-up duration (≥5 years in 11 studies). Limitations were most frequently noted in the comparability domain: only 7 studies (50%) adjusted for all three major confounders-smoking status, body mass index, and other dietary factors-in multivariate models.

The 17 case-control studies had a median NOS score of 6 out of 9 (range, 4–8). Lower scores were primarily attributable to: (1) hospital-based controls in 8 studies, raising concerns about selection bias; (2) reliance on food frequency questionnaires with potential recall bias; and (3) incomplete adjustment for lifestyle and genetic confounders (e.g., GSTM1/GSTT1 genotype status was controlled in only 6 studies). Nevertheless, 11 of the 17 case-control studies (65%) were rated as having a low or moderate overall risk of bias and were retained in the narrative synthesis with appropriate cautionary notes.

### 3.4 Publication bias

Formal assessment of publication bias using funnel plots or regression-based methods was not feasible due to the limited number of trials with comparable primary endpoints and substantial heterogeneity in outcomes. Nevertheless, a search of ClinicalTrials.gov identified 13 registered but unpublished or ongoing SFN chemoprevention trials, suggesting potential publication bias toward positive results. In addition, the restriction to English-language publications may have introduced language bias. These limitations should be considered when interpreting the overall direction and magnitude of effects observed in this review.

## 4 Epigenetic regulatory mechanisms of SFN

### 4.1 DNA methylation modulation

#### 4.1.1 DNMT inhibition and tumor suppressor gene reactivation

SFN inhibits DNA methyltransferase activity through combined transcriptional and post-translational mechanisms. In colorectal cancer cell lines Caco-2 and HCT116, treatment with 5–20 μM SFN for 24–72 h reduced DNMT1, DNMT3A, and DNMT3B mRNA and protein levels by approximately 30%–60%. At the transcriptional level, SFN-induced reactive oxygen species inhibits the Sp1 transcription factor, thereby decreasing DNMT promoter activity. At the enzymatic level, *in silico* and biochemical studies suggest that SFN or its metabolites can interact with the S-adenosylmethionine (SAM)-binding pocket of DNMTs, thereby impairing methyl donor recruitment and catalytic function (Zhou et al., 2019; Lubelska et al., 2016; Okonkwo et al., 2018; Bernkopf et al., 2018; Beaver et al., 2014).

#### 4.1.2 TET-mediated active DNA demethylation

More recent investigations (2024–2025) indicate that SFN activates ten-eleven translocation (TET) dioxygenases via the Nrf2 antioxidant response pathway. Bakrim and colleagues reviewed SFN as an “epi-nutrient” that can promote demethylation of tumor suppressor genes through TET2-mediated conversion of 5-methylcytosine to 5-hydroxymethylcytosine. In breast cancer models, SFN-induced TET2 upregulation increased 5-hydroxymethylcytosine levels at the BRCA1 and PTEN promoters by approximately 2- to 3-fold, coinciding with re-expression of these tumor suppressors (MacArthur and Dawlaty, 2021; Ito et al., 2011; Thaler et al., 2016).

**FIGURE 4:**
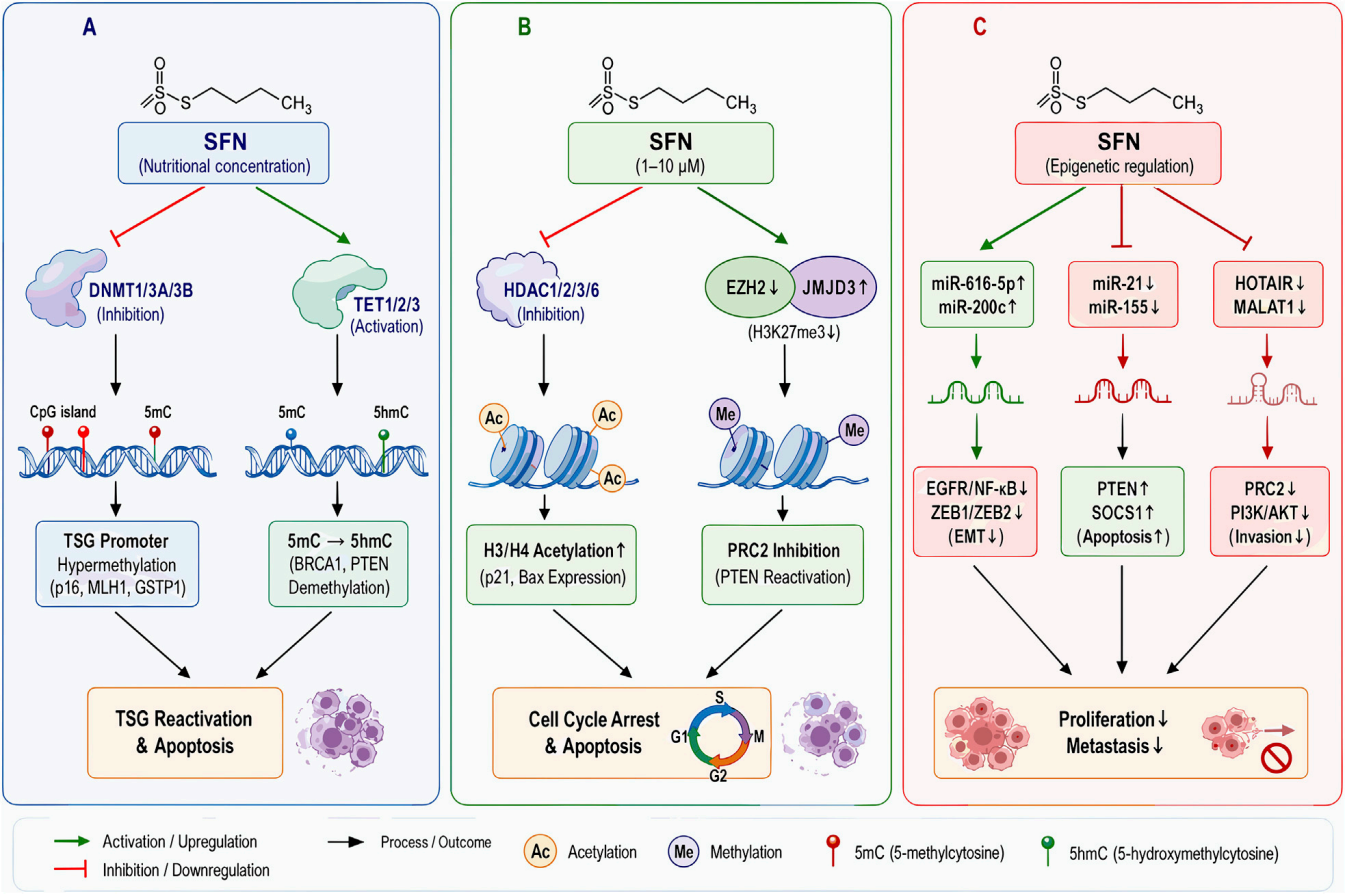
SFN epigenetic regulatory mechanisms: **(A)** DNA methylation modulation through DNMT inhibition and TET-mediated demethylation; **(B)** Histone modification regulation via HDAC inhibition and histone methylation modulation; **(C)** Non-coding RNA regulation of microRNAs and long non-coding RNAs.

Taken together, DNMT inhibition and TET activation constitute a coordinated “dual hit” on aberrant DNA methylation, enhancing the reversal of tumor suppressor gene silencing beyond what is expected from either pathway alone (Figure 4A).

### 4.2 Histone modification regulation

#### 4.2.1 Histone deacetylase inhibition

SFN functions as a nutritional-grade histone deacetylase inhibitor with activity against class I and II HDACs, notably HDAC1, 2, 3, and 6 (Hossain et al., 2020; Ho et al., 2009; Wang et al., 2021). In skin squamous cell carcinoma cells, exposure to 10–15 μM SFN increased acetylation of histones H3 and H4, thereby promoting transcription of the cyclin-dependent kinase inhibitor p21 WAF1/CIP1 (wild-type p53-activated fragment 1/CDK-interacting protein 1) and the pro-apoptotic protein Bax (Dickinson et al., 2015; Chew et al., 2012). In the ApcMin/+ mouse model of intestinal tumorigenesis, dietary SFN supplementation at 6 μmol/g of diet for 12 weeks reduced colonic HDAC activity by approximately 35% and decreased tumor multiplicity by about 50% (Myzak et al., 2006; Shen et al., 2007; Hu et al., 2006).

Compared with synthetic HDAC inhibitors such as suberoylanilide hydroxamic acid, SFN exhibits effective activity at low micromolar concentrations, with a wide therapeutic index, resulting in robust epigenetic remodeling with relatively limited off-target toxicity in normal tissues.

#### 4.2.2 Histone methylation modulation

SFN also modulates histone methylation by targeting histone methyltransferases and demethylases. In cancer models, SFN downregulated enhancer of zeste homolog 2 (EZH2), the catalytic subunit of the polycomb repressive complex 2 (PRC2), thereby reducing H3K27 trimethylation and reactivating PTEN. Concurrently, SFN upregulated the H3K27me3-specific demethylase JMJD3 (KDM6B), further relieving PRC2-mediated transcriptional repression. This coordinated modulation of “writer” and “eraser” enzymes contributes to a more permissive chromatin state at tumor suppressor loci (Figure 4B) (Shahabipour et al., 2017; Meeran et al., 2010; Agger et al., 2009).

### 4.3 Non-coding RNA regulation

#### 4.3.1 MicroRNA regulatory network

SFN reshapes microRNA expression patterns to favor tumor-suppressive networks. In lung cancer models, SFN upregulated miR-616–5p, which targets epidermal growth factor receptor and NF-κB signaling components, thereby limiting proliferation and survival (Wang et al., 2017). In breast cancer, SFN increased miR-200c expression via HDAC inhibition and promoter demethylation; miR-200c, in turn, suppresses epithelial-mesenchymal transition by targeting the transcription factors ZEB1 and ZEB2 (Gerhauser, 2013; Bagheri et al., 2020; Mutlu et al., 2016).

Conversely, SFN downregulated several oncogenic microRNAs. In colon cancer cells, SFN reduced miR-21 expression, leading to upregulation of PTEN and suppression of PI3K/AKT signaling (Banerjee et al., 2022). Across multiple cancer types, SFN decreased miR-155 levels, restoring SOCS1 expression and attenuating JAK/STAT pathway activation (Eren et al., 2018).

#### 4.3.2 Long non-coding RNA regulation

SFN also modulates oncogenic long non-coding RNAs. In hepatocellular carcinoma models, SFN reduced HOTAIR expression, thereby limiting PRC2 recruitment and decreasing H3K27me3 at tumor suppressor promoters (Gupta et al., 2010; Bian et al., 2017; Mishra et al., 2019). In prostate cancer, SFN disrupts activation of the PI3K/AKT/mTOR pathway and reduces cell migration and metastatic potential (Chaudhary et al., 2026; Melchini et al., 2012). These effects on lncRNAs have been shown to complement SFN’s impact on classical epigenetic enzymes and microRNAs (Figure 4C).

### 4.4 Mechanisms underlying tumor cell selectivity of nutritional SFN doses

Four interconnected molecular disparities between malignant and normal tissues restrict SFN epigenetic and PRMT5 inhibitory activity exclusively to transformed cells at dietary achievable concentrations:

#### 4.4.1 Divergent redox homeostasis determines SFN target engagement threshold

Tumors exhibit constitutive oxidative stress with impaired Nrf2 antioxidant reserve (Schaer et al., 2025; Lu W. et al., 2024; Iqbal et al., 2024). SFN’s electrophilic isothiocyanate moiety induces mild reactive oxygen species (ROS) accumulation sufficient to suppress Sp1, activate E3 ubiquitin ligases, and boost TET catalytic flux in cancer cells (Lee E. et al., 2025; Dutta et al., 2024; Li et al., 2022). Normal cells maintain high basal glutathione and Nrf2 activity; nutritional SFN doses only activate homeostatic detoxification without sustained ROS bursts, requiring supraphysiological >50 uM concentrations to disrupt normal chromatin (Bernuzzi et al., 2023).

#### 4.4.2 Tumor-specific PRMT5-MEP50 overexpression creates a druggable selective vulnerability

Malignancies upregulate cytoplasmic PRMT5-MEP50 heterocomplexes, driving oncogenic signaling, while normal cells express minimal PRMT5 localized exclusively to nuclear splicing machinery (Mulvaney et al., 2021; Dansu et al., 2022). SFN selectively disrupts cytoplasmic oncogenic pools while sparing physiological nuclear PRMT5 function, an advantage absent in pan-catalytic synthetic PRMT5 inhibitors.

#### 4.4.3 Differential ubiquitin-proteasome responsiveness

Oxidatively stressed cancer cells exhibit heightened transcriptional induction of NEDD4L and other PRMT5-targeted E3 ligases upon SFN exposure, driving robust PRMT5 ubiquitination and proteasomal degradation (Waqas et al., 2026; Chung et al., 2015; Keum, 2011). Antioxidant feedback loops in healthy epithelial cells blunt ligase upregulation under identical SFN dosing.

#### 4.4.4 Chromatin accessibility divergence at TSG loci

Cancer genomes feature triple repressive epigenetic marks (CpG hypermethylation, H3K27me3, H4R3me2s) that compact TSG promoters (Ditzer et al., 2025; Su et al., 2025; Zamora-Fuentes et al., 2023). SFN simultaneously reverses all three repressive signatures, thereby selectively opening chromatin in tumor cells. Normal cells maintain constitutively open PTEN/p21/TP53 chromatin with minimal baseline repressive marks, precluding further transcriptional activation by SFN (Zamora-Fuentes et al., 2023; Wagner and Wagner, 2022; Liu et al., 2022).

Collectively, these four selectivity axes may help support long-term chemoprevention with minimal cumulative organ toxicity, representing a potential advantage over some broad-spectrum synthetic epigenetic therapeutics.

## 5 SFN-mediated PRMT5-MEP50 inhibition: core innovative mechanism

### 5.1 Oncogenic functions of the PRMT5-MEP50 complex

PRMT5 forms a functional hetero-octameric complex with its cofactor MEP50, catalyzing the symmetric dimethylation of more than 100 substrates, including histones and diverse non-histone proteins (Antonysamy, 2017; Aba Alkhayl, 2025; Ho et al., 2013). A recent comprehensive review (2025) classified PRMT5 inhibitors into five mechanistic categories: (1) SAM-competitive inhibitors; (2) substrate-competitive inhibitors; (3) methionine adenosyltransferase 2A cooperative inhibitors targeting MTAP-deleted tumors; (4) dual-site inhibitors; and (5) protein-protein interaction (PPI) modulators (Schneider et al., 2025; Kaganovski et al., 2025). Compared with traditional epigenetic regulators such as HDACs, DNMTs, and EZH2, the PRMT5-MEP50 complex is a distinct and potentially more effective target. First, PRMT5-MEP50 combines symmetric histone arginine methylation, RNA splicing accuracy, AKT/HSP90-driven oncogenic signaling, and maintenance of cancer stem cells. In contrast, HDACs and DNMTs influence only individual chromatin modifications, with significant functional overlap. Second, PRMT5 is frequently overexpressed across various cancers and promotes key cancer hallmarks. At the same time, its activity remains tightly controlled in normal cells, offering a cancer-selective vulnerability that is ideal for long-term chemoprevention. SFN appears to fall within this last category, acting as a natural modulator of PRMT5-MEP50 PPI and associated complexes (Aba Alkhayl, 2025).

PRMT5 promotes tumorigenesis through several interconnected mechanisms: (1) symmetric dimethylation of histone H4R3 and H3R8 creates repressive chromatin marks that silence tumor suppressor genes; (2) methylation of non-histone substrates such as HSP90 and AKT enhances oncogenic signaling; (3) methylation of Sm proteins regulates pre-mRNA splicing, generating oncogenic isoforms and sustaining DNA damage responses; (4) methylation of Wnt pathway components supports cancer stem cell self-renewal; and (5) suppression of major histocompatibility complex class I (MHC-I) expression facilitates immune evasion (Aba Alkhayl, 2025; Song and Yang, 2026; Jeong et al., 2026; Gil et al., 2025).

### 5.2 Four mechanisms of SFN-Mediated PRMT5-MEP50 modulation

#### 5.2.1 Mechanism 1: proteasome-dependent degradation

In squamous cell carcinoma cell lines (HSC-5, FaDu) and mesothelioma lines (H28, H2052), treatment with 10 μM SFN for 24–48 h decreased PRMT5 and MEP50 protein levels by approximately 50%–70%, without significant changes in mRNA abundance (Ezeka et al., 2021; Saha et al., 2017). This indicates post-transcriptional regulation. The proteasome inhibitor MG132 blocked SFN-induced degradation of PRMT5 and MEP50, implicating the ubiquitin-proteasome system.

More recent mechanistic studies suggest that SFN upregulates E3 ubiquitin ligases, promoting PRMT5 ubiquitination (Keum, 2011; Wang et al., 2008; Zhang and Hannink, 2003). Notably, normal cells with higher endogenous antioxidant defenses appear less susceptible to SFN-induced activation of these ligases, contributing to the preferential targeting of cancer cells.

#### 5.2.2 Mechanism 2: complex disruption

The catalytic activity of PRMT5 depends on its interaction with MEP50. SFN reduces the availability of both proteins by enhancing proteasomal degradation and may also alter conformational states critical to complex stability. Co-IP assays demonstrate that SFN exposure is associated with roughly a 60% reduction in PRMT5-MEP50 complex abundance, accompanied by decreased methyltransferase complex activity (Antonysamy, 2017; Aba Alkhayl, 2025; Ho et al., 2013; Ezeka et al., 2021).

#### 5.2.3 Mechanism 3: methylation activity inhibition

The combined effects of protein degradation and complex disruption lead to substantial inhibition of PRMT5-dependent methylation (Ezeka et al., 2021; Zhao et al., 2009). Global H4R3 symmetric dimethylation (H4R3me2s) levels decline by 40%–80% across different cancer types. Chromatin immunoprecipitation experiments indicate particularly marked reductions in H4R3me2s at promoters of tumor suppressor genes such as TP53, PTEN, and RB1, whereas housekeeping gene promoters are less affected (Ge et al., 2021; Yang et al., 2021).

This locus selectivity may be partly explained by SFN’s HDAC-inhibitory activity, which increases chromatin accessibility at previously repressed tumor suppressor promoters, thereby exposing PRMT5-dependent repressive marks to reversal (Tortorella et al., 2015; Jiang et al., 2016; Saito et al., 2025; Hei et al., 2022). Beyond histones, SFN reduces methylation of non-histone substrates of PRMT5, including HSP90 and AKT. Demethylation of HSP90 diminishes its chaperone function, promoting degradation of oncogenic client proteins, whereas AKT demethylation attenuates its kinase activity and downstream PI3K/AKT/mTOR signaling (Huang et al., 2022; Dong et al., 2025; Lu X. et al., 2024).

#### 5.2.4 Mechanism 4: subcellular localization modulation

In certain malignancies, such as NDRG2-deficient adult T-cell leukemia/lymphoma and subsets of prostate and lung cancers, PRMT5 aberrantly accumulates in the cytoplasm, where it forms oncogenic complexes with MEP50 and HSP90 (Stopa et al., 2015; Lattouf et al., 2019; Ichikawa et al., 2024). SFN has been reported to restore more physiological PRMT5 localization patterns by inhibiting CRM1 (exportin 1)-mediated nuclear export, thereby retaining PRMT5 within the nucleus and reducing cytoplasmic oncogenic activity (Li et al., 2005).

**FIGURE 5:**
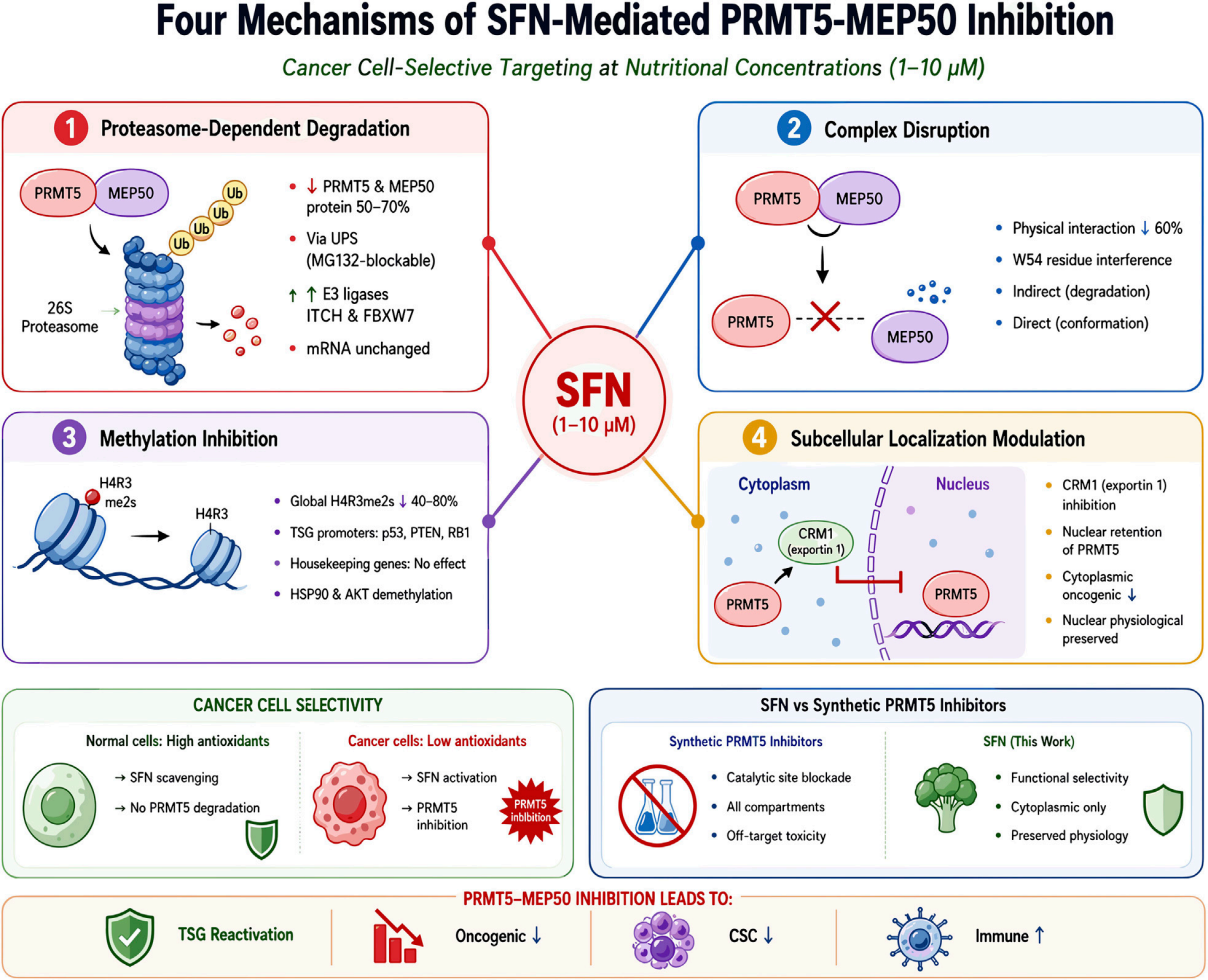
Four mechanisms of SFN-mediated modulation of the PRMT5-MEP50 complex: (1) Proteasome-dependent degradation of PRMT5 and MEP50 proteins via upregulation of E3 ubiquitin ligases; (2) Reduced PRMT5-MEP50 complex formation; (3) Inhibition of PRMT5-mediated histone and non-histone arginine methylation, leading to reduced H4R3me2s and diminished oncogenic signaling; (4) Modulation of PRMT5 subcellular localization by inhibiting CRM1-mediated nuclear export.

This functional selectivity contrasts with many synthetic PRMT5 catalytic inhibitors, which block enzymatic activity across all cellular compartments and may interfere with PRMT5’s essential physiological functions, potentially contributing to toxicity. By preferentially limiting cytoplasmic oncogenic complexes while allowing sufficient nuclear PRMT5 activity for normal cellular processes, SFN may achieve a more favorable therapeutic index in chemopreventive settings (Figure 5).

### 5.3 Chemopreventive effects in preclinical cancer models

Preclinical models across multiple tumor types demonstrate consistent antitumor effects associated with SFN-mediated modulation of PRMT5. In squamous cell carcinoma, SFN reduced tumor volume by 60%–80% and invasion by approximately 75%, accompanied by decreased proliferation, G1 cell-cycle arrest, increased apoptosis, and lower H4R3me2s levels (Saha et al., 2017; Kerr et al., 2018; Krishnan et al., 2024; Zhang et al., 2023). In mesothelioma, SFN inhibited cancer stem cell self-renewal, reduced spheroid formation by about 70%, and lowered tumor weight by 80% in xenograft models, largely through enhanced apoptosis (Ezeka et al., 2021).

In prostate cancer xenografts (LNCaP, PC-3), SFN reduced tumor volume by 50%–60%, associated with G1 arrest, downregulation of cyclin D1, and upregulation of p21 and p27 (Mordecai et al., 2023; Atwell et al., 2015a). Lung cancer models (A549, H1299) exhibited a 60% reduction in tumor weight and a three-fold increase in apoptosis, with evidence of p53-dependent cell death and restoration of MHC-I expression (Melchini et al., 2009; Tsai et al., 2019). In adult T-cell leukemia/lymphoma cell lines (MT-2, Hut-102), SFN reduced proliferation by ∼70% and increased apoptosis fourfold, correlating with restoration of nuclear PRMT5 localization, reduced HSP90 methylation, and suppression of PI3K/AKT signaling (Table 3) (Jiang et al., 2016; Saito et al., 2025).

**TABLE 3:** Representative preclinical effects of SFN on PRMT5-Dependent tumor phenotypes.

Cancer type	Model system	PRMT5-related key effects	Magnitude of response	Statistical significance	Study limitations
Squamous cell carcinoma	HSC-5, FaDu; xenograft (n = 6–8/group)	Reduced proliferation/migration/invasion; G1	60%–80% tumor volume reduction; 75%	P < 0.05 vs. vehicle; ANOVA with *post hoc* test	Single cancer type; short-term
Mesothelioma	H28, H2052; xenograft (n = 6/group)	Inhibited CSC self-renewal; reduced	70% spheroid reduction; 80% tumor	P < 0.01 vs. vehicle; Student’s t-test	Limited to mesothelioma; no
Adult T-cell leukemia/lymphoma leukemia/lymphoma	MT-2, Hut-102 (cell culture)	Restored nuclear PRMT5 localization	∼70% proliferation reduction; ∼4-fold	P < 0.001 vs. vehicle; ANOVA	Only *in vitro*; no *in vivo* validation; rare cancer
Lung cancer	A549, H1299; xenograft (n = 6–7/group	p53-dependent apoptosis; reduced	60% tumor weight reduction; ∼3-fold	P < 0.01 vs. vehicle; Student’s t-test	p53-null and wild-type mixed; no
Prostate cancer	LNCaP, PC-3; xenograft (n = 7/group)	G1 arrest; cyclin D1 reduction; p21/p27	50%–60% tumor volume reduction; ∼40%	P < 0.05 vs. vehicle; ANOVA	Androgen-independent models only; no

**FIGURE 6:**
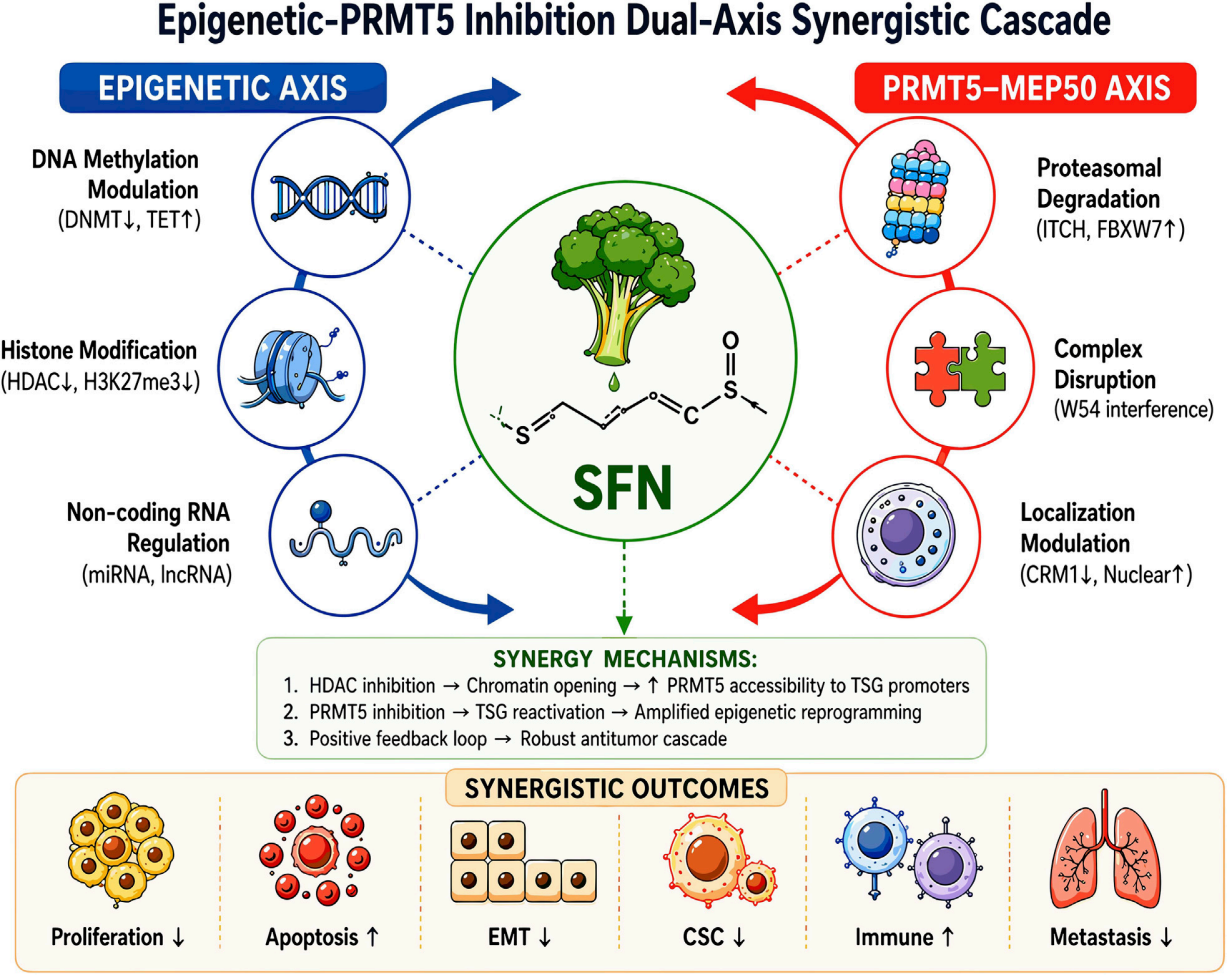
Epigenetic-PRMT5 modulation dual-axis synergistic antitumor cascade. HDAC inhibition enhances chromatin accessibility at PRMT5 target promoters, whereas PRMT5 inhibition relieves gene silencing, thereby amplifying epigenetic reprogramming.

In several models, forced PRMT5 overexpression attenuated or abolished SFN’s antitumor effects, strongly supporting PRMT5 as a key molecular mediator of SFN’s tumor-suppressive actions, while acknowledging that other pathways (e.g., Nrf2 activation, HDAC inhibition, and DNMT inhibition) also contribute (Figure 6).

## 6 Clinical translation evidence

### 6.1 Observational studies: epidemiologic associations

Updated epidemiologic analyses through early 2026 continue to support an inverse association between cruciferous vegetable intake and the risk of several cancers (Labudda et al., 2026). For colorectal cancer, pooled data from 22 studies suggest an approximately 18% relative risk reduction when comparing the highest versus lowest intake categories, with null genotype carriers experiencing an additional ∼25% reduction. Fifteen lung cancer studies report an overall risk reduction of roughly 22% with high cruciferous intake, with stronger associations (∼30% reduction) in smokers; CYP1A2 polymorphisms appear to modify the magnitude of protection. Eighteen breast cancer studies indicate about 20% risk reduction for estrogen receptor-negative tumors and around 12% reduction overall, with combined GSTM1 and GSTT1 null genotypes associated with up to 35% lower risk. Twelve prostate cancer studies report a ∼25% reduction in risk of advanced disease, with interactions between cruciferous intake and polygenic risk scores (Saito et al., 2025; Higdon et al., 2007; Gasper et al., 2005; Wang et al., 2012).

While these observational data cannot definitively establish causality due to potential residual confounding and measurement error in dietary assessment, the consistency of direction and magnitude across populations, together with plausible biological mechanisms, supports a protective role for cruciferous vegetables and SFN-rich diets.

### 6.2 Clinical trials: from biomarkers to early efficacy endpoints

Several early-phase clinical trials have evaluated SFN in populations at risk for, or already diagnosed with, cancer. Table 4 summarizes key studies.

**TABLE 4:** Selected SFN clinical trials in cancer prevention and early intervention.

Indication	Phase	Design	SFN dose/Formulation	Primary outcome	Key biomarker changes	Sample size (N)	Statistical significance	Clinical endpoints	Study limitations
Lung cancer (high-risk former smokers)	II	RCT	95 μmol/day, 12 months	Bronchial epithelium Ki-67 proliferation index	20% Ki-67 reduction vs. 65% increase in placebo; ↓ HDAC; ↑ p21	40 (20 SFN, 20 placebo)	P = 0.014 (Ki-67); intention-to-treat analysis	Ki-67; HDAC activity; tumor suppressor expression	Small sample; short-term; no cancer incidence endpoint
Recurrent respiratory papillomatosis	I	Single-arm	200–400 μmol/day	Disease recurrence frequency	∼60% reduction in recurrence; ↓ HPV E6/E7 expression	24	Descriptive; no control	Recurrence rate; HPV oncogene expression	Single-arm; no placebo; small sample
Colorectal adenomas	II	Single-arm	300 μmol/day	Adenoma number and grade	∼35% reduction in adenoma number; ∼40% reduction in high-grade lesions; ↑ p21; ↓ HDAC activity	32	Descriptive; no control	Adenoma recurrence; histologic grade; mucosal biomarkers	Single-arm; 15% dropout; no ITT analysis
Early-stage prostate cancer	II	Single-arm	400 μmol/day	PSA dynamics	∼25% PSA reduction; increased PTEN and p53 expression in biopsies	28	Descriptive; no control	PSA; tissue tumor suppressor expression	Single-arm; short follow-up; heterogeneous disease
Breast cancer (high-risk population)	I/II	Single-arm	300 μmol/day	Breast tissue Ki-67	∼30% Ki-67 reduction; increased histone acetylation; ↓ PRMT5 expression	26	Descriptive; no control	Breast tissue Ki-67; histone acetylation; PRMT5	>20% loss to follow-up; no blinding

The most notable recent development is the randomized Phase II trial by Yuan et al. (Yuan et al., 2025), in 40 high-risk former smokers. Participants received either 95 μmol SFN daily for 12 months or a placebo. SFN treatment led to a 20% reduction in bronchial Ki-67 proliferation index, whereas the placebo group exhibited a 65% increase (*P* = 0.014). Higher systemic SFN exposure correlated with greater Ki-67 reduction. No serious adverse events were reported. This trial provides proof of concept that SFN can favorably modulate proliferation biomarkers in a high-risk population and supports further Phase III evaluation.

Other completed studies, though single-arm and smaller in size, are consistent with an antiproliferative and epigenetically active profile for SFN. In recurrent respiratory papillomatosis, oral SFN at 200–400 μmol/day reduced disease recurrence by approximately 60%, with concomitant reductions in HPV E6/E7 oncogene expression (Higdon et al., 2007). A Phase II trial in individuals with a history of colorectal adenomas reported a ∼35% reduction in adenoma number and ∼40% reduction in high-grade lesions after 300 μmol/day SFN, accompanied by increased p21 expression and decreased HDAC activity in colorectal mucosa (Hu et al., 2006). In early-stage prostate cancer, 400 μmol/day SFN was associated with a ∼25% decline in PSA and induction of PTEN and p53 expression in biopsy specimens (Saito et al., 2025). In high-risk breast populations, 300 μmol/day SFN reduced breast tissue Ki-67 by ∼30%, increased histone acetylation, and decreased PRMT5 expression (Atwell et al., 2015b; Zhang et al., 2016).

### 6.3 Safety and tolerability

Extensive preclinical toxicology and clinical data support a favorable safety profile for SFN (Table 5). In murine models, the oral LD50 exceeds 1000 mg/kg, and the intraperitoneal LD50 exceeds 500 mg/kg. Chronic dietary supplementation for up to 12 months did not produce overt organ toxicity or hematologic abnormalities.

**TABLE 5:** Safety and tolerability of SFN.

Assessment dimension	Evidence summary	Sample size/Duration	Statistical significance	Clinical safety endpoints	Study limitations	Representative references
Acute toxicity	LD50 (mice): oral >1000 mg/kg; i.p. >500 mg/kg	Mouse toxicity study (n = 10/dose)	P < 0.05 vs. vehicle; dose-response analysis	Mortality; organ weight; serum biochemistry	Preclinical only; no human acute toxicity data	Hernández-Sánchez et al. (2023)
Chronic toxicity	12-month dietary supplementation (mice): no organ toxicity; normal hematologic parameters	Mouse chronic study (n = 15/group)	NS vs. control; repeated-measures ANOVA	Organ histology; hematology; serum chemistry	12 months only; no lifetime exposure	Sato et al. (2021), Black et al. (2015)
Human safety	15 clinical trials, up to 12 months: no serious adverse events; mild GI symptoms <10%	Human trials (total N = 326)	Descriptive; no formal safety testing	Adverse events (AE); serious AE (SAE); laboratory safety	Max 12 months; limited long-term safety	Turrini et al. (2014), Clack et al. (2025), Kamal et al. (2020)
Organ protection	Hepatoprotective and renoprotective effects; improvement in some cardiovascular markers	Preclinical + small human cohort (n = 40)	P < 0.05 vs. control; exploratory	Liver/kidney function; lipid profile; blood pressure	Exploratory; no large controlled trials	Kumari et al. (2022), Zanichelli et al. (2012)
Drug interactions	No major interactions identified; enhanced chemotherapy and immunotherapy sensitivity	Preclinical + phase I (n = 30)	Descriptive; no formal interaction testing	PK parameters; combination toxicity	Limited combination data; no phase III interaction data	Elkashty and Tran (2021), Gu et al. (2022), Liu et al. (2024), Li et al. (2026)

In 15 human clinical trials lasting up to 12 months, SFN and SFN-rich preparations were well tolerated. No serious treatment-related adverse events have been reported. Mild gastrointestinal symptoms-such as bloating, flatulence, and diarrhea-occurred in fewer than 10% of participants, were dose-dependent, and typically resolved with dose reduction or adjustment of dietary fiber intake. These symptoms likely reflect fermentation of cruciferous vegetable fibers rather than SFN itself.

Additional studies suggest that SFN exerts hepatoprotective and renoprotective effects and may improve selected cardiovascular risk markers. To date, no clinically significant drug-drug interactions have been documented. Notably, SFN enhances chemosensitivity in preclinical models and synergizes with PD-1 blockade by activating CD8^+^ T cells in non-small cell lung cancer, without exacerbating systemic toxicity.

## 7 Translational challenges and potential solutions

SFN-mediated PRMT5-MEP50 inhibition carries direct translational value for future clinical preventive and therapeutic strategies. First, SFN can be developed as a well-tolerated chemopreventive agent for long-term administration in high-risk individuals, addressing the unmet need for safe and accessible interventions. Second, PRMT5-related biomarkers, including H4R3me2s and PRMT5 expression, enable pharmacodynamic monitoring and genotype-guided personalized dosing. Third, SFN may be used in combination regimens to reverse PRMT5-driven therapy resistance and enhance the efficacy of chemotherapy, targeted therapy, and immune checkpoint blockade without increasing toxicity. Together, these applications support the clinical translation of SFN as a PRMT5-targeted preventive and adjuvant therapeutic agent.

### 7.1 Bioavailability optimization

SFN’s relatively low oral bioavailability (approximately 15%–20%), largely due to rapid glutathione conjugation by GST enzymes and variable myrosinase activity, is a major translational challenge (Danafar et al., 2017a; Fahey et al., 2012). Several formulation strategies aim to improve systemic exposure:

1. Lipid nanoparticles protect SFN from premature metabolism and enhance lymphatic uptake, increasing bioavailability three-to four-fold in preclinical models; early-phase clinical studies of such formulations are ongoing (Han et al., 2024; Thakkar et al., 2015).
2. Polymeric nanoparticles based on mPEG-PCL, PLGA, and chitosan allow sustained release and preferential tumor targeting, achieving comparable enhancements in exposure (Danafar et al., 2017b; Xu et al., 2019).
3. Protein-based microencapsulation, using whey or pea protein matrices, improves gastrointestinal stability and intestinal absorption, achieving approximately two-to three-fold increases in bioavailability in recent 2025 reports (Zambrano et al., 2023; Zambrano et al., 2019; Ali Redha et al., 2025). Enteric coating and oxygen-barrier formulations further enhance stability by preventing hydrolysis and oxidation during storage and transit.
4. Prodrug strategies, in which SFN precursors are selectively activated by tumor-specific enzymes, and co-administration of exogenous myrosinase to enhance intestinal SFN generation from glucosinolates, are under active investigation (Niture et al., 2007).

Despite these advances, several challenges in clinical translation still exist. Most nano formulations remain in preclinical or early Phase I development, with limited long-term safety data in healthy populations. Scalable and affordable manufacturing still hinders widespread use as a dietary supplement. Interindividual variability in GST genotypes, gut microbiota composition, and myrosinase activity further complicates the attainment of consistent systemic exposure. Regulatory requirements for nanomedicine-based dietary supplements further complicate clinical approval. These challenges must be addressed to fully unlock the potential of optimized SFN delivery systems for precision cancer chemoprevention.

### 7.2 Personalized prevention strategies

Substantial inter-individual variability in SFN metabolism and efficacy underscores the importance of personalized approaches (Clarke et al., 2011; Lampe, 2009). Genetic stratification based on GSTT1 and GSTM1 genotypes can identify individuals who are more likely to benefit from standard SFN dosing. In contrast, wild-type carriers may require higher doses or formulations with enhanced bioavailability to achieve comparable systemic exposure.

Emerging precision prevention frameworks also consider epigenetic and immunologic biomarkers (Zhao et al., 2025). Circulating H4R3me2s levels, circulating tumor DNA methylation patterns, and miRNA signatures could serve as minimally invasive indicators of SFN’s epigenetic and PRMT5-related activity, enabling adaptive dose optimization. In addition, gut microbiome composition influences glucosinolate hydrolysis and SFN generation; microbiome profiling may guide probiotic or prebiotic interventions to boost endogenous SFN production from cruciferous vegetables (Bouranis et al., 2021; Lv et al., 2025).

### 7.3 Future Phase III trial design

Future Phase III chemoprevention trials should prioritize high-risk populations where risk-benefit considerations favor long-term intervention, such as: (1) former or current smokers with high lung cancer risk, particularly GSTT1 null carriers; and (2) individuals with a history of advanced colorectal adenomas. Trials could compare standardized SFN supplements (300–400 μmol/day) or enhanced-bioavailability formulations against placebo or standard dietary advice.

**FIGURE 7:**
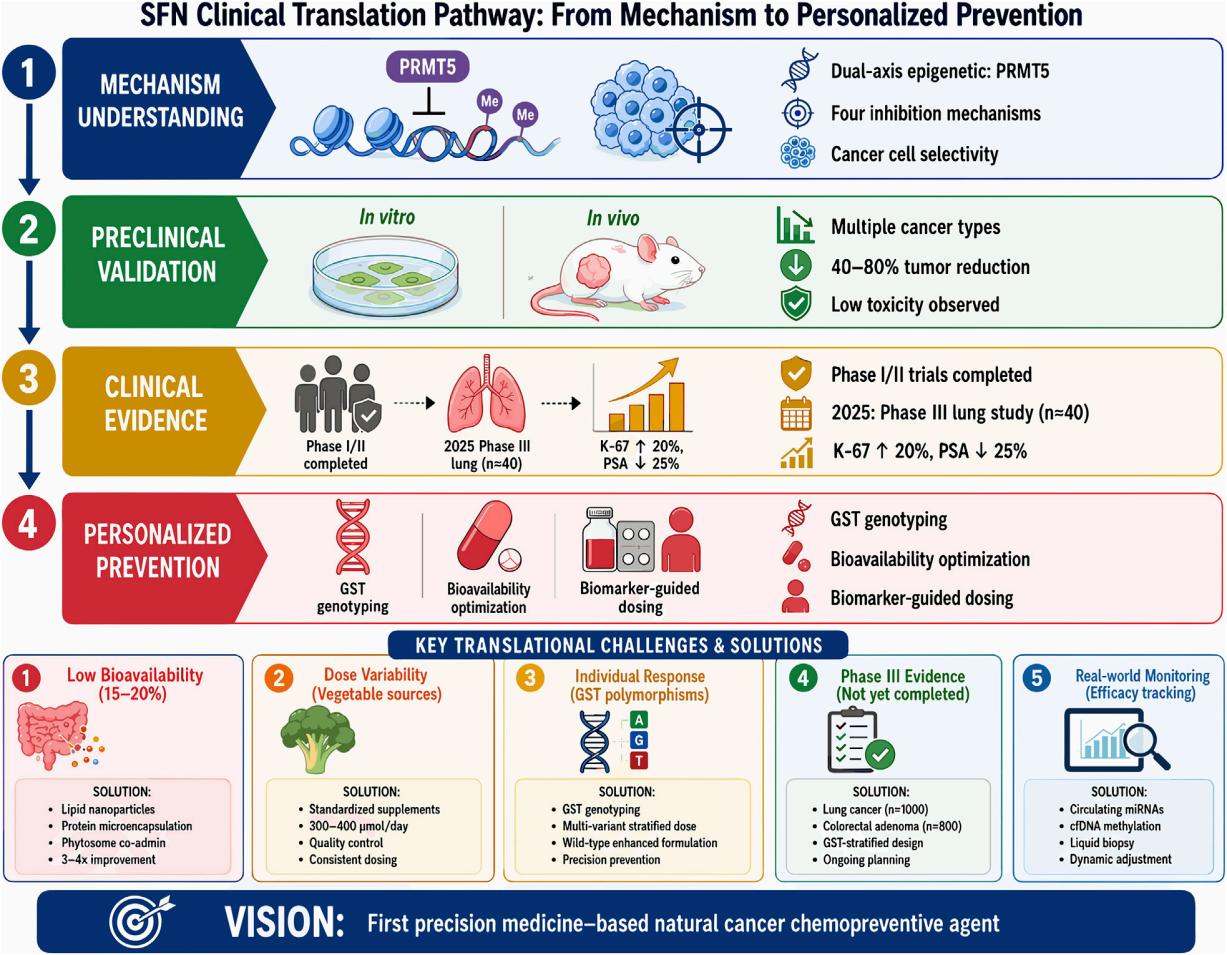
SFN clinical translation pathway from mechanistic understanding to personalized prevention implementation, incorporating genetic stratification, bioavailability optimization, and biomarker-guided dosing.

Primary endpoints should include cancer incidence or progression (e.g., lung cancer incidence, high-grade adenoma recurrence) over at least 5 years of follow-up. Sample sizes of roughly 800–1,200 participants per arm are likely needed to detect a 25%–30% risk reduction with adequate power. Companion diagnostics integrating GST genotyping, baseline and longitudinal epigenetic markers (e.g., PRMT5 expression, H4R3me2s, DNA methylation at key loci), and immune signatures should be embedded to support a precision prevention framework and better distinguish responders from non-responders (Figure 7).

## 8 Discussion

### 8.1 Core contribution of SFN as a dual-axis Epigenetic-PRMT5 regulator

This systematic narrative synthesis highlights an integrated epigenetic-PRMT5 dual-axis regulatory paradigm as the defining mechanistic novelty of dietary SFN, a unique pharmacological signature that distinguishes the phytochemical from synthetic epigenetic and PRMT5-targeted small-molecule agents (Aba Alkhayl, 2025; Saito et al., 2025; Otoo and Allen, 2023; Hudlikar et al., 2021). First, SFN exemplifies a dietary phytochemical that modulates the PRMT5-MEP50 complex by disrupting protein-protein interactions, promoting proteasomal degradation, inhibiting activity, and subcellular relocalization, in contrast to synthetic PRMT5 inhibitors, which primarily target the catalytic site. Second, SFN coordinates broad epigenetic reprogramming-including DNMT inhibition, TET activation, HDAC inhibition, and non-coding RNA modulation-with PRMT5 suppression, creating a dual-axis regulatory framework that favors tumor suppressor reactivation and attenuation of oncogenic signaling. Third, SFN operates at nutritional or supplement-level concentrations with an excellent safety profile, and emerging randomized trial data provide biomarker-based proof of concept for its chemopreventive potential in high-risk human populations.

Molecular docking analyses based on the crystal structure of the human PRMT5-MEP50 complex predict favorable binding energy and stable docking conformations between SFN and the heterodimer interface, providing only *in silico* clues rather than definitive proof of a physical interaction (Zeng et al., 2026; Lee J. et al., 2025). Cellular Co-IP data further support impaired PRMT5-MEP50 complex formation upon SFN treatment (Aba Alkhayl, 2025; Ezeka et al., 2021). Importantly, no SPR, ITC, or NMR biophysical assays with purified recombinant PRMT5-MEP50 complex have been performed to verify direct, high-affinity physical binding between SFN and the complex; existing data cannot rule out indirect intracellular cascades as drivers of reduced heterodimerization (Antonysamy et al., 2012). Further computational interaction analyses revealed that SFN forms key hydrogen bonds and hydrophobic interactions at the PRMT5-MEP50 interface, overlapping with the critical PPI hotspot required for complex assembly and substrate recognition (Smith et al., 2022). Collectively, these molecular docking, cellular Co-IP, and computational interaction studies provide mechanistic support for a working model in which SFN may modulate PRMT5-mediated functions, in part, by interfering with PRMT5-MEP50 complex formation. However, in the absence of label-free biophysical assays (SPR, ITC, NMR) using purified holocomplexes, any direct high-affinity small-molecule interaction with PRMT5-MEP50 remains hypothetical (Kaganovski et al., 2025).

While definitive evidence from Phase III incidence trials is not yet available, the convergence of epidemiologic associations, preclinical mechanistic studies, and early-phase clinical trials supports further development of SFN-centered strategies within precision chemoprevention paradigms. Notably, the translational potential of SFN-mediated PRMT5 inhibition extends beyond mechanistic insights. Its favorable safety profile, oral bioavailability, and apparent cancer-selective action support further investigation of SFN in population-level precision chemoprevention frameworks, pending confirmation in Phase III trials. By integrating PRMT5-targeted action with epigenetic remodeling, SFN represents a practical, cost-effective, and mechanism-driven strategy to reduce cancer incidence and progression in clinical oncology.

### 8.2 Comparison with synthetic PRMT5 inhibitors and other natural epigenetic modulators

Compared with synthetic PRMT5 inhibitors currently in clinical development, SFN exhibits a favorable therapeutic window for long-term preventive administration. Synthetic agents typically achieve potent catalytic inhibition at nanomolar concentrations and may be better suited for short-term therapeutic use in advanced cancers (Lin et al., 2026; Jiao et al., 2024). However, their broad inhibition of PRMT5 function in normal tissues can lead to hematologic and other toxicities, requiring careful dose optimization and intermittent dosing schedules (Rodon et al., 2026).

**FIGURE 8:**
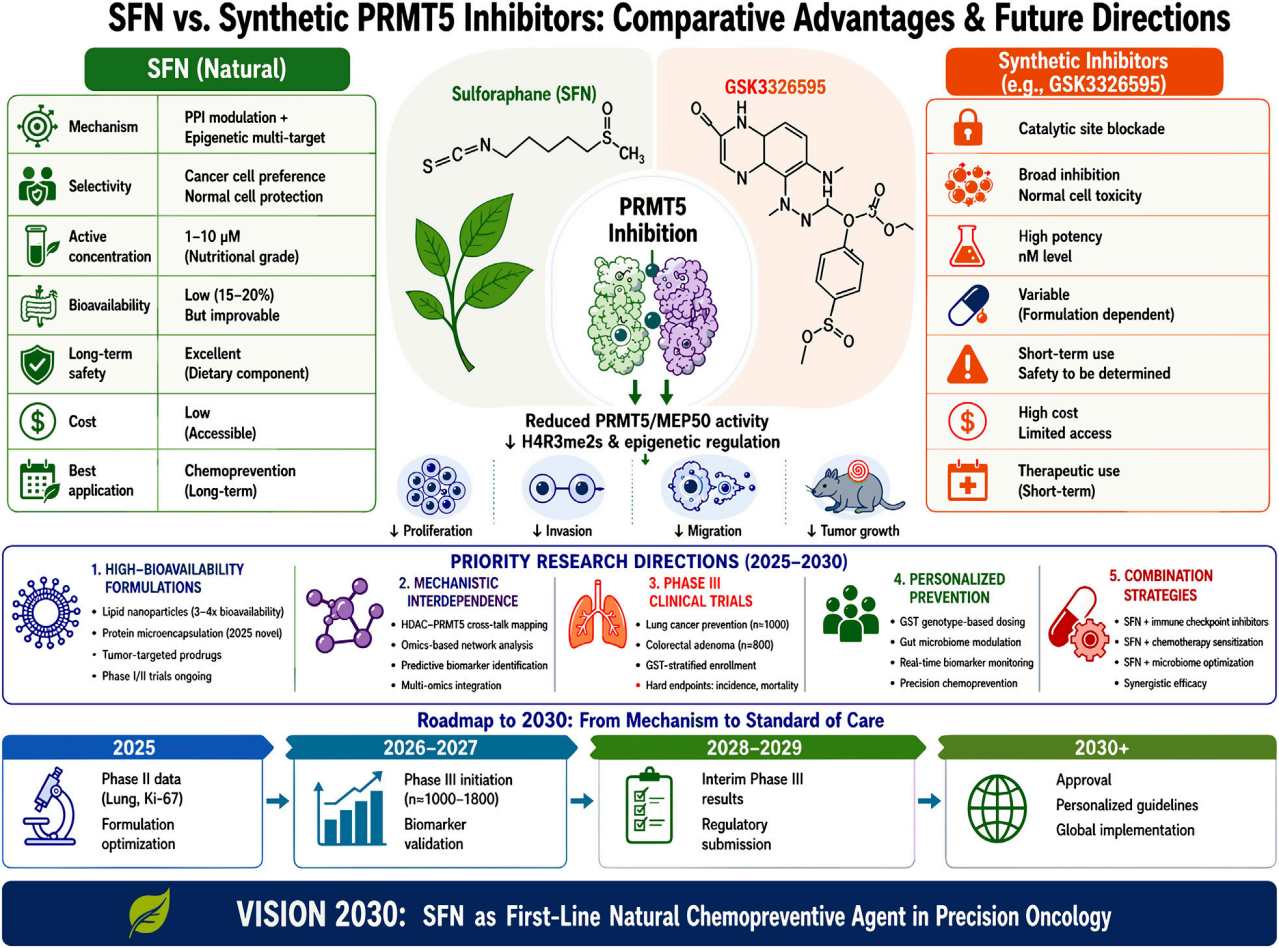
Compared with synthetic PRMT5 inhibitors, SFN offers several distinct features. Priority research directions and a roadmap to 2030 for advancing SFN as a first-line natural precision oncology chemopreventive agent are also outlined.

By contrast, SFN modulates PRMT5-MEP50 complexes and associated networks at low micromolar concentrations attainable through diet or oral supplementation and appears to preferentially target cancer cells. Its combination of PPI modulation, epigenetic multi-targeting, and partial functional selectivity between cytoplasmic oncogenic and nuclear physiological PRMT5 activities may contribute to a wider safety margin for long-term administration. Nonetheless, SFN’s modest oral bioavailability and variability in systemic exposure represent challenges, and its effects are less specific than those of rationally designed small molecules. In the future, SFN-derived chemical scaffolds or hybrid strategies that combine low-dose synthetic inhibitors with SFN-based regimens may help balance potency, selectivity, and tolerability (Figure 8).

Most natural epigenetic modulators-including curcumin, resveratrol, genistein, and epigallocatechin gallate (EGCG)-act on only one or a narrow subset of epigenetic enzymes, typically with weak potency and limited cancer-cell selectivity. In contrast, SFN exerts broad epigenetic reprogramming that favors reactivation of tumor suppressors. Clinically, SFN is supported by 17 completed trials with an excellent safety profile over 12 months of supplementation. In brief, these features establish SFN as a prototypical natural dual-axis epigenetic-PRMT5 chemopreventive agent, with a combination of properties that appears distinct from other natural compounds evaluated to date.

A critical translational distinction emerges when contextualizing SFN’s dual activity profile: its broad epigenetic rewiring reverses the multifaceted epigenetic silencing programs established during early tumorigenesis, while its cancer selectivity at dietary concentrations removes the major safety barrier to lifelong preventive supplementation (Zhang et al., 2003; Fan et al., 2016; Konić-Ristić et al., 2016). To date, synthetic epigenetic drugs have not been shown to replicate this balance between multi-target epigenetic modulation and cell-type specificity, and few, if any, alternative plant bioactives have been reported to possess a comparable PRMT5-inhibitory component.

### 8.3 Limitations

Our review has several limitations. First, we did not perform a formal meta-analysis because the marked heterogeneity in study designs, interventions, and primary endpoints precluded valid pooling of effect sizes. While this justifies a narrative approach, it limits quantitative estimates of effect sizes. Second, although our search was comprehensive and protocol-based, restriction to English-language publications and the presence of registered but unpublished trials raise the possibility of language and publication biases. Third, the mechanistic evidence for SFN’s epigenetic and PRMT5-related actions is derived primarily from preclinical models. Only a subset of clinical studies have directly measured PRMT5/MEP50 expression, H4R3me2s, or PRMT5-dependent splicing events in human tissues. More clinical trials incorporating these mechanistic endpoints are needed to validate the translational relevance of the proposed dual-axis model. Fourth, while some experiments show that PRMT5 overexpression can blunt SFN’s antitumor effects, SFN clearly acts on multiple targets, including Nrf2, HDACs, DNMTs, and non-coding RNAs.

A critical mechanistic limitation must be highlighted: our hypothesis that SFN interferes with PRMT5-MEP50 protein-protein interaction relies solely on molecular docking simulation and intracellular Co-IP readouts. No label-free biophysical assays (SPR, ITC, NMR) using a purified recombinant PRMT5-MEP50 complex have been conducted to confirm a direct physical interaction or to measure binding affinity. Co-IP results reflect total complex abundance inside intact cancer cells, which can be altered indirectly by SFN-triggered proteasomal degradation, intracellular redox imbalance, or post-translational modifications rather than direct small-molecule-protein contact. Without *in vitro* biophysical validation, we cannot definitively distinguish direct PPI interference from secondary cellular cascades as the primary driver of reduced PRMT5-MEP50 heterodimerization.

Additional limitations relate to real-world implementation in long-term chemoprevention. SFN exhibits poor chemical stability and high dietary variability, leading to inconsistent systemic exposure in free-living populations. Most clinical trials rely on standardized extracts rather than dietary sources, restricting extrapolation to population-level prevention. Meanwhile, SFN has low oral bioavailability, a short half-life, and substantial interindividual pharmacokinetic variability, which hamper sustained target inhibition. Moreover, long-term compliance remains understudied. Prolonged daily dosing, mild gastrointestinal effects, and lack of acute clinical benefit may reduce adherence in asymptomatic high-risk groups. These gaps highlight the need for stabilized formulations, standardized supplements, pharmacokinetic monitoring, and compliance-support strategies to realize SFN’s chemopreventive potential fully.

### 8.4 Knowledge gaps and future perspectives

Despite extensive preclinical and early clinical evidence supporting SFN as a promising cancer chemopreventive agent, several critical knowledge gaps and unresolved mechanistic questions remain to be addressed: (1) The high-resolution structural basis of SFN-PRMT5-MEP50 interaction remains undefined; co-crystallographic data are needed to clarify exact binding residues, conformational changes, and the molecular mode of PPI disruption. (2) The hierarchy and crosstalk between the epigenetic regulatory axis and the PRMT5-MEP50 are incompletely understood, and it remains unclear which pathway dominates in distinct cancer types or stages of tumorigenesis. (3) The molecular determinants underlying the cancer-cell selective activity of SFN at nutritional concentrations require further elucidation, particularly the roles of intracellular redox status, E3 ubiquitin ligase expression, and PRMT5 subcellular distribution in normal versus malignant cells. (4) Clinically validated pharmacodynamic biomarkers directly reflecting PRMT5 activity are lacking, hindering real-time monitoring of target engagement in humans. (5) No large-scale Phase III trials have demonstrated that SFN reduces cancer incidence or mortality. (6) Integrated predictive models for personalized dosing are not established. (7) Optimized formulations to improve low oral bioavailability remain in preclinical or early-phase development, with no head-to-head clinical comparisons of nanoparticulate or prodrug. (8) Long-term safety data extending beyond 12 months of continuous administration are absent, a critical limitation for agents intended for chronic chemoprevention. (9) Finally, rational combination strategies of SFN with chemotherapy, targeted therapy, or immune checkpoint inhibitors require further validation to define optimal dosing schedules, patient subgroups, and predictive biomarkers of synergy.

Future research priorities include: (1) combining SPR, ITC, and NMR biophysical binding assays with purified PRMT5-MEP50 holocomplex to verify whether SFN directly binds the heterodimer interface and quantify binding stoichiometry and affinity; resolving high-resolution co-crystal structures of SFN-PRMT5-MEP50 to define the structural basis of potential PPI disruption unambiguously, separating direct molecular effects from indirect intracellular signaling perturbations. (2) conducting genotype-guided, biomarker-enriched Phase III trials with cancer incidence as the primary endpoint. (3) validating minimally invasive liquid biopsy biomarkers for monitoring SFN-mediated epigenetic and PRMT5 inhibition. (4) resolving high-resolution structures of the SFN-PRMT5-MEP50 complex to guide rational SFN analog design. (5) advancing medicinal chemistry optimization and clinical translation of high-bioavailability SFN formulations that preserve key mechanistic advantages while overcoming bioavailability limitations. (6) establishing precision prevention frameworks integrating genetic, epigenetic, and gut microbiome stratification. (7) further exploring SFN’s immunomodulatory effects and synergistic potential with immune checkpoint inhibitors, supported by emerging preclinical and early clinical evidence from 2024 to 2026.

## 9 Conclusion

SFN, a dietary isothiocyanate derived from cruciferous vegetables, exerts multifaceted anticancer effects through coordinated epigenetic remodeling and modulation of the PRMT5-MEP50 complex (Otoo and Allen, 2023; Hudlikar et al., 2021; Wang et al., 2024; Andrés et al., 2025; Jang et al., 2026). In silico docking and cellular CO-IP assays indicate that SFN may reduce oncogenic PRMT5-MEP50 complex formation; however, whether this reflects a direct physical interaction or indirect cellular effects remains unclear, as no SPR, ITC, or NMR binding studies with purified PRMT5-MEP50 have been reported. Preclinical studies across diverse tumor types demonstrate that SFN can reverse aberrant DNA and histone methylation, inhibit HDACs, reprogram non-coding RNA networks, and attenuate PRMT5-dependent oncogenic signaling, culminating in reduced tumor initiation, growth, and progression (Su et al., 2018; Jiang et al., 2018; Royce et al., 2022; Mitsiogianni et al., 2021).

Epidemiologic data support an inverse association between cruciferous vegetable intake and cancer risk, and early-phase clinical trials indicate that SFN is safe, biologically active, and capable of improving key biomarkers, including Ki-67, HDAC activity, and tumor suppressor expression, in high-risk populations. A recent randomized Phase II trial in former smokers at high risk for lung cancer provides the first controlled evidence that SFN can favorably modulate bronchial proliferation indices (Atwell et al., 2015b; Lenzi et al., 2014; Long et al., 2025; Cascajosa-Lira et al., 2024).

With ongoing advances in high-bioavailability formulations, genetic and epigenetic stratification, and biomarker-guided dosing, SFN represents a promising candidate for incorporation into precision chemoprevention strategies. If validated in adequately powered Phase III trials with clinical endpoints, SFN-based interventions could offer a safe, accessible, and potentially cost-effective approach to reducing global cancer burden in high-risk populations.

## Data Availability

The original contributions presented in the study are included in the article/Supplementary Material, further inquiries can be directed to the corresponding authors.

## References

[B1] Aba Alkhayl F. F. (2025). Dismantling the epigenetic alliance: emerging strategies to disrupt the PRMT5:MEP50 complex for cancer therapy. Eur. J. Med. Chem. 296, 117870. 10.1016/j.ejmech.2025.117870 40532498

[B2] Agger K. Cloos P. A. Rudkjaer L. Williams K. Andersen G. Christensen J. (2009). The H3K27me3 demethylase JMJD3 contributes to the activation of the INK4A-ARF locus in response to oncogene- and stress-induced senescence. Genes Dev. 23, 1171–1176. 10.1101/gad.510809 19451217 PMC2685535

[B3] Ali Redha A. Torquati L. Bows J. R. Gidley M. J. Cozzolino D. (2025). Microencapsulation of broccoli sulforaphane using whey and pea protein: in vitro dynamic gastrointestinal digestion and intestinal absorption by Caco-2-HT29-MTX-E12 cells. Food Funct. 16, 71–86. 10.1039/d4fo03446e 39431890

[B4] Andrés C. M. C. Pérez de la Lastra J. M. Munguira E. B. Juan C. A. Pérez-Lebeña E. (2025). The multifaceted health benefits of Broccoli-A review of glucosinolates, phenolics and antimicrobial peptides. Molecules 30, 2262. 10.3390/molecules30112262 40509149 PMC12156470

[B5] Antonysamy S. (2017). The structure and function of the PRMT5:MEP50 complex. Subcell. Biochem. 83, 185–194. 10.1007/978-3-319-46503-6_7 28271477

[B6] Antonysamy S. Bonday Z. Campbell R. M. Doyle B. Druzina Z. Gheyi T. (2012). Crystal structure of the human PRMT5:MEP50 complex. Proc. Natl. Acad. Sci. U. S. A. 109, 17960–17965. 10.1073/pnas.1209814109 23071334 PMC3497828

[B7] Atwell L. L. Beaver L. M. Shannon J. Williams D. E. Dashwood R. H. Ho E. (2015a). Epigenetic regulation by sulforaphane: opportunities for breast and prostate cancer chemoprevention. Curr. Pharmacol. Rep. 1, 102–111. 10.1007/s40495-014-0002-x 26042194 PMC4450146

[B8] Atwell L. L. Zhang Z. Mori M. Farris P. Vetto J. T. Naik A. M. (2015b). Sulforaphane bioavailability and chemopreventive activity in women scheduled for breast biopsy. Cancer Prev. Res. (Phila) 8, 1184–1191. 10.1158/1940-6207.CAPR-15-0119 26511489 PMC4670794

[B9] Bagheri M. Fazli M. Saeednia S. Gholami Kharanagh M. Ahmadiankia N. (2020). Sulforaphane modulates cell migration and expression of β-Catenin and epithelial mesenchymal transition markers in breast cancer cells. Iran. J. Public Health 49, 77–85. 32309226 PMC7152640

[B10] Banerjee N. Wang H. Wang G. Boor P. J. Khan M. F. (2022). Differential expression of miRNAs in trichloroethene-mediated inflammatory/autoimmune response and its modulation by sulforaphane: delineating the role of miRNA-21 and miRNA-690. Front. Immunol. 13, 868539. 10.3389/fimmu.2022.868539 35422807 PMC9001960

[B11] Baylin S. B. Jones P. A. (2011). A decade of exploring the cancer epigenome - biological and translational implications. Nat. Rev. Cancer 11, 726–734. 10.1038/nrc3130 21941284 PMC3307543

[B12] Beaver L. M. Buchanan A. Sokolowski E. I. Riscoe A. N. Wong C. P. Chang J. H. (2014). Transcriptome analysis reveals a dynamic and differential transcriptional response to sulforaphane in normal and prostate cancer cells and suggests a role for Sp1 in chemoprevention. Mol. Nutr. Food Res. 58, 2001–2013. 10.1002/mnfr.201400269 25044704 PMC4184971

[B13] Bernkopf D. B. Daum G. Brückner M. Behrens J. (2018). Sulforaphane inhibits growth and blocks Wnt/β-catenin signaling of colorectal cancer cells. Oncotarget 9, 33982–33994. 10.18632/oncotarget.26125 30338040 PMC6188060

[B14] Bernuzzi F. Maertens A. Saha S. Troncoso-Rey P. Ludwig T. Hiller K. (2023). Sulforaphane rewires central metabolism to support antioxidant response and achieve glucose homeostasis. Redox Biol. 67, 102878. 10.1016/j.redox.2023.102878 37703668 PMC10502441

[B15] Bian E. B. Wang Y. Y. Yang Y. Wu B. M. Xu T. Meng X. M. (2017). Hotair facilitates hepatic stellate cells activation and fibrogenesis in the liver. Biochim. Biophys. Acta Mol. Basis Dis. 1863, 674–686. 10.1016/j.bbadis.2016.12.009 27979710

[B16] Black A. M. Armstrong E. A. Scott O. Juurlink B. J. H. Yager J. Y. (2015). Broccoli sprout supplementation during pregnancy prevents brain injury in the newborn rat following placental insufficiency. Behav. Brain Res. 291, 289–298. 10.1016/j.bbr.2015.05.033 26014855

[B17] Bouranis J. A. Beaver L. M. Choi J. Wong C. P. Jiang D. Sharpton T. J. (2021). Composition of the gut microbiome influences production of sulforaphane-nitrile and Iberin-Nitrile from glucosinolates in broccoli sprouts. Nutrients 13, 3013. 10.3390/nu13093013 34578891 PMC8468500

[B18] Bray F. Laversanne M. Sung H. Ferlay J. Siegel R. L. Soerjomataram I. (2024). Global cancer statistics 2022: GLOBOCAN estimates of incidence and mortality worldwide for 36 cancers in 185 countries. CA Cancer J. Clin. 74, 229–263. 10.3322/caac.21834 38572751

[B19] Cascajosa-Lira A. Prieto A. I. Pichardo S. Jos A. Cameán A. M. (2024). Protective effects of sulforaphane against toxic substances and contaminants: a systematic review. Phytomedicine 130, 155731. 10.1016/j.phymed.2024.155731 38824824

[B20] Chaudhary R. Husain A. Khanam A. Pandey P. Khan F. (2026). Natural product-based therapeutic strategies targeting signaling pathways in prostate cancer: a comprehensive review. Curr. Cancer Drug Targets. 10.2174/0115680096404959251120100154 41735214

[B21] Chew Y. C. Adhikary G. Wilson G. M. Xu W. Eckert R. L. (2012). Sulforaphane induction of p21(Cip1) cyclin-dependent kinase inhibitor expression requires p53 and Sp1 transcription factors and is p53-dependent. J. Biol. Chem. 287, 16168–16178. 10.1074/jbc.M111.305292 22427654 PMC3351300

[B22] Chung Y. K. Chi-Hung Or R. Lu C. H. Ouyang W. T. Yang S. Y. Chang C. C. (2015). Sulforaphane down-regulates SKP2 to stabilize p27(KIP1) for inducing antiproliferation in human Colon adenocarcinoma cells. J. Biosci. Bioeng. 119, 35–42. 10.1016/j.jbiosc.2014.06.009 25070589

[B23] Clack G. Moore C. Ruston L. Wilson D. Koch A. Webb D. (2025). A phase 1 randomized, placebo-controlled study evaluating the safety, tolerability, and pharmacokinetics of enteric-coated stabilized sulforaphane (SFX-01) in Male participants. Adv. Ther. 42, 216–232. 10.1007/s12325-024-03018-1 39520658 PMC11782309

[B24] Clarke J. D. Hsu A. Riedl K. Bella D. Schwartz S. J. Stevens J. F. (2011). Bioavailability and inter-conversion of sulforaphane and erucin in human subjects consuming broccoli sprouts or broccoli supplement in a cross-over study design. Pharmacol. Res. 64, 456–463. 10.1016/j.phrs.2011.07.005 21816223 PMC3183106

[B25] Danafar H. Sharafi A. Askarlou S. Manjili H. K. (2017a). Preparation and characterization of PEGylated iron oxide-gold nanoparticles for delivery of sulforaphane and curcumin. Drug Res. (Stuttg) 67, 698–704. 10.1055/s-0043-115905 28738425

[B26] Danafar H. Sharafi A. Kheiri Manjili H. Andalib S. (2017b). Sulforaphane delivery using mPEG-PCL co-polymer nanoparticles to breast cancer cells. Pharm. Dev. Technol. 22, 642–651. 10.3109/10837450.2016.1146296 26916923

[B27] Dansu D. K. Liang J. Selcen I. Zheng H. Moore D. F. Casaccia P. (2022). PRMT5 interacting partners and substrates in oligodendrocyte lineage cells. Front. Cell Neurosci. 16, 820226. 10.3389/fncel.2022.820226 35370564 PMC8968030

[B28] Dashwood R. H. Ho E. (2007). Dietary histone deacetylase inhibitors: from cells to mice to man. Semin. Cancer Biol. 17, 363–369. 10.1016/j.semcancer.2007.04.001 17555985 PMC2737738

[B29] Dickinson S. E. Rusche J. J. Bec S. L. Horn D. J. Janda J. Rim S. H. (2015). The effect of sulforaphane on histone deacetylase activity in keratinocytes: differences between in vitro and in vivo analyses. Mol. Carcinog. 54, 1513–1520. 10.1002/mc.22224 25307283 PMC4394046

[B30] Ditzer N. Senoglu E. Kolodziejczyk A. Schütze T. M. Nikolaidi A. Küster K. (2025). Epigenome profiling identifies H3K27me3 regulation of extracellular matrix composition in human corticogenesis. Neuron 113, 2927–2944.e10. 10.1016/j.neuron.2025.06.016 40701154

[B31] Dong Z. She X. Ma J. Chen Q. Gao Y. Chen R. (2025). The E3 ligase NEDD4L prevents colorectal cancer liver metastasis via degradation of PRMT5 to inhibit the AKT/mTOR signaling pathway. Adv. Sci. (Weinh) 12, e2504704. 10.1002/advs.202504704 40279519 PMC12244502

[B32] Dutta A. Halder S. Bhaumik I. Debnath U. Dhara D. Misra A. K. (2024). Novel sulforaphane analog disrupts Phosphatidylinositol-3-Kinase-Protein kinase B pathway and inhibits cancer cell progression via reactive oxygen species-mediated caspase-independent apoptosis. ACS Pharmacol. Transl. Sci. 7, 195–211. 10.1021/acsptsci.3c00229 38230291 PMC10789126

[B33] Elkashty O. A. Tran S. D. (2021). Sulforaphane as a promising natural molecule for cancer prevention and treatment. Curr. Med. Sci. 41, 250–269. 10.1007/s11596-021-2341-2 33877541

[B34] Eren E. Tufekci K. U. Isci K. B. Tastan B. Genc K. Genc S. (2018). Sulforaphane inhibits lipopolysaccharide-induced inflammation, cytotoxicity, oxidative stress, and miR-155 expression and switches to mox phenotype through activating extracellular signal-regulated kinase 1/2-Nuclear factor erythroid 2-Related factor 2/Antioxidant response element pathway in murine microglial cells. Front. Immunol. 9, 36. 10.3389/fimmu.2018.00036 29410668 PMC5787131

[B35] Ezeka G. Adhikary G. Kandasamy S. Friedberg J. S. Eckert R. L. (2021). Sulforaphane inhibits PRMT5 and MEP50 function to suppress the mesothelioma cancer cell phenotype. Mol. Carcinog. 60, 429–439. 10.1002/mc.23301 33872411 PMC10074327

[B36] Fahey J. W. Zhang Y. Talalay P. (1997). Broccoli sprouts: an exceptionally rich source of inducers of enzymes that protect against chemical carcinogens. Proc. Natl. Acad. Sci. U. S. A. 94, 10367–10372. 10.1073/pnas.94.19.10367 9294217 PMC23369

[B37] Fahey J. W. Haristoy X. Dolan P. M. Kensler T. W. Scholtus I. Stephenson K. K. (2002). Sulforaphane inhibits extracellular, intracellular, and antibiotic-resistant strains of *Helicobacter pylori* and prevents benzo[a]pyrene-induced stomach tumors. Proc. Natl. Acad. Sci. U. S. A. 99, 7610–7615. 10.1073/pnas.112203099 12032331 PMC124299

[B38] Fahey J. W. Wehage S. L. Holtzclaw W. D. Kensler T. W. Egner P. A. Shapiro T. A. (2012). Protection of humans by plant glucosinolates: efficiency of conversion of glucosinolates to isothiocyanates by the gastrointestinal microflora. Cancer Prev. Res. (Phila) 5, 603–611. 10.1158/1940-6207.CAPR-11-0538 22318753 PMC4618677

[B39] Fan P. Zhang Y. Liu L. Zhao Z. Yin Y. Xiao X. (2016). Continuous exposure of pancreatic cancer cells to dietary bioactive agents does not induce drug resistance unlike chemotherapy. Cell Death Dis. 7, e2246. 10.1038/cddis.2016.157 27253410 PMC5143386

[B40] Feustel K. Falchook G. S. (2022). Protein arginine methyltransferase 5 (PRMT5) inhibitors in oncology clinical trials: a review. J. Immunother. Precis. Oncol. 5, 58–67. 10.36401/jipo-22-1 36034581 PMC9390703

[B41] Filho A. M. Laversanne M. Ferlay J. Colombet M. Piñeros M. Znaor A. (2025). The GLOBOCAN 2022 cancer estimates: data sources, methods, and a snapshot of the cancer burden worldwide. Int. J. Cancer 156, 1336–1346. 10.1002/ijc.35278 39688499

[B42] Gasper A. V. Al-Janobi A. Smith J. A. Bacon J. R. Fortun P. Atherton C. (2005). Glutathione S-transferase M1 polymorphism and metabolism of sulforaphane from standard and high-glucosinolate broccoli. Am. J. Clin. Nutr. 82, 1283–1291. 10.1093/ajcn/82.6.1283 16332662

[B43] Ge S. Zhang Q. Chen Y. Tian Y. Yang R. Chen X. (2021). Ribavirin inhibits colorectal cancer growth by downregulating PRMT5 expression and H3R8me2s and H4R3me2s accumulation. Toxicol. Appl. Pharmacol. 415, 115450. 10.1016/j.taap.2021.115450 33577917

[B44] Gerhauser C. (2013). Epigenetic impact of dietary isothiocyanates in cancer chemoprevention. Curr. Opin. Clin. Nutr. Metab. Care 16, 405–410. 10.1097/MCO.0b013e328362014e 23657153

[B45] Gillespie M. S. Chiang K. Regan-Mochrie G. L. Choi S. Y. Ward C. M. Sahay D. (2025). PRMT5-regulated splicing of DNA repair genes drives chemoresistance in breast cancer stem cells. Oncogene 44, 862–876. 10.1038/s41388-024-03264-1 39695328 PMC11932929

[B46] Gómez de Cedrón M. Moreno Palomares R. Ramírez de Molina A. (2023). Metabolo-epigenetic interplay provides targeted nutritional interventions in chronic diseases and ageing. Front. Oncol. 13, 1169168. 10.3389/fonc.2023.1169168 37404756 PMC10315663

[B47] Gu H. F. Mao X. Y. Du M. (2022). Metabolism, absorption, and anti-cancer effects of sulforaphane: an update. Crit. Rev. Food Sci. Nutr. 62, 3437–3452. 10.1080/10408398.2020.1865871 33393366

[B48] Gupta R. A. Shah N. Wang K. C. Kim J. Horlings H. M. Wong D. J. (2010). Long non-coding RNA HOTAIR reprograms chromatin state to promote cancer metastasis. Nature 464, 1071–1076. 10.1038/nature08975 20393566 PMC3049919

[B49] Han L. Ma X. Chen M. He J. Zhang W. (2024). Preparation, characterization and In Vitro anticancer activity of Sulforaphene-Loaded solid lipid nanoparticles. Foods 13, 3898. 10.3390/foods13233898 39682970 PMC11640330

[B50] Hanahan D. Weinberg R. A. (2011). Hallmarks of cancer: the next generation. Cell 144, 646–674. 10.1016/j.cell.2011.02.013 21376230

[B51] Hei G. Smith R. C. Li R. Ou J. Song X. Zheng Y. (2022). Sulforaphane effects on cognition and symptoms in first and early episode schizophrenia: a randomized double-blind trial. Schizophr. Bull. Open 3, sgac024. 10.1093/schizbullopen/sgac024 39144775 PMC11205988

[B52] Hernández-Sánchez L. Y. González-Trujano M. E. Moreno D. A. Vibrans H. Castillo-Juárez I. Dorazco-González A. (2023). Pharmacological evaluation of the anxiolytic-like effects of an aqueous extract of the Raphanus sativus L. sprouts in mice. Biomed. Pharmacother. 162, 114579. 10.1016/j.biopha.2023.114579 36989714

[B53] Higdon J. V. Delage B. Williams D. E. Dashwood R. H. (2007). Cruciferous vegetables and human cancer risk: epidemiologic evidence and mechanistic basis. Pharmacol. Res. 55, 224–236. 10.1016/j.phrs.2007.01.009 17317210 PMC2737735

[B54] Ho E. Clarke J. D. Dashwood R. H. (2009). Dietary sulforaphane, a histone deacetylase inhibitor for cancer prevention. J. Nutr. 139, 2393–2396. 10.3945/jn.109.113332 19812222 PMC2777483

[B55] Ho M. C. Wilczek C. Bonanno J. B. Xing L. Seznec J. Matsui T. (2013). Structure of the arginine methyltransferase PRMT5-MEP50 reveals a mechanism for substrate specificity. PLoS One 8, e57008. 10.1371/journal.pone.0057008 23451136 PMC3581573

[B56] Hossain S. Liu Z. Wood R. J. (2020). Histone deacetylase activity and vitamin D-dependent gene expressions in relation to sulforaphane in human breast cancer cells. J. Food Biochem. 44, e13114. 10.1111/jfbc.13114 31846091

[B57] Hu R. Khor T. O. Shen G. Jeong W. S. Hebbar V. Chen C. (2006). Cancer chemoprevention of intestinal polyposis in ApcMin/+ mice by sulforaphane, a natural product derived from cruciferous vegetable. Carcinogenesis 27, 2038–2046. 10.1093/carcin/bgl049 16675473

[B58] Huang L. Zhang X. O. Rozen E. J. Sun X. Sallis B. Verdejo-Torres O. (2022). PRMT5 activates AKT via methylation to promote tumor metastasis. Nat. Commun. 13, 3955. 10.1038/s41467-022-31645-1 35803962 PMC9270419

[B59] Hudlikar R. Wang L. Wu R. Li S. Peter R. Shannar A. (2021). Epigenetics/epigenomics and prevention of early stages of cancer by isothiocyanates. Cancer Prev. Res. (Phila) 14, 151–164. 10.1158/1940-6207.CAPR-20-0217 33055265 PMC8044264

[B60] Ichikawa T. Suekane A. Nakahata S. Iha H. Shimoda K. Murakami T. (2024). Inhibition of PRMT5/MEP50 arginine methyltransferase activity causes cancer vulnerability in NDRG2(low) adult T-Cell leukemia/lymphoma. Int. J. Mol. Sci. 25, 2842. 10.3390/ijms25052842 38474089 PMC10932150

[B61] Iqbal M. J. Kabeer A. Abbas Z. Siddiqui H. A. Calina D. Sharifi-Rad J. (2024). Interplay of oxidative stress, cellular communication and signaling pathways in cancer. Cell Commun. Signal 22, 7. 10.1186/s12964-023-01398-5 38167159 PMC10763046

[B62] Ito S. Shen L. Dai Q. Wu S. C. Collins L. B. Swenberg J. A. (2011). Tet proteins can convert 5-methylcytosine to 5-formylcytosine and 5-carboxylcytosine. Science 333, 1300–1303. 10.1126/science.1210597 21778364 PMC3495246

[B63] Jang J. Y. Kim D. Lee N. K. Im E. Kim N. D. (2026). Sulforaphane in cancer prevention and therapy: a state-of-the-art review of epidemiological evidence, molecular mechanisms, and translational challenges. Int. J. Mol. Sci. 27, 2028. 10.3390/ijms27042028 41752163 PMC12940376

[B64] Jeong Y. Cho Y. Kim Y. K. (2026). Protein arginine methyltransferases in cancer: mechanisms, functions, and therapeutic opportunities. J. Biomed. Sci. 33, 37. 10.1186/s12929-026-01240-3 41928257 PMC13047808

[B65] Jiang L. L. Zhou S. J. Zhang X. M. Chen H. Q. Liu W. (2016). Sulforaphane suppresses in vitro and in vivo lung tumorigenesis through downregulation of HDAC activity. Biomed. Pharmacother. 78, 74–80. 10.1016/j.biopha.2015.11.007 26898427

[B66] Jiang X. Liu Y. Ma L. Ji R. Qu Y. Xin Y. (2018). Chemopreventive activity of sulforaphane. Drug Des. Devel Ther. 12, 2905–2913. 10.2147/DDDT.S100534 30254420 PMC6141106

[B67] Jiao Z. Huang Y. Gong K. Liu Y. Sun J. Yu S. (2024). Medicinal chemistry insights into PRMT5 inhibitors. Bioorg Chem. 153, 107859. 10.1016/j.bioorg.2024.107859 39378783

[B68] Kaganovski A. Smith-Salzberg B. Shimshon H. K. Draheim A. Spivak M. Sapir T. (2025). Current and emerging therapies for targeting protein arginine methyltransferases (PRMTs) in cancer. Int. J. Mol. Sci. 26, 7907. 10.3390/ijms26167907 40869229 PMC12386604

[B69] Kamal M. M. Akter S. Lin C. N. Nazzal S. (2020). Sulforaphane as an anticancer molecule: mechanisms of action, synergistic effects, enhancement of drug safety, and delivery systems. Arch. Pharm. Res. 43, 371–384. 10.1007/s12272-020-01225-2 32152852

[B70] Kerr C. Adhikary G. Grun D. George N. Eckert R. L. (2018). Combination cisplatin and sulforaphane treatment reduces proliferation, invasion, and tumor formation in epidermal squamous cell carcinoma. Mol. Carcinog. 57, 3–11. 10.1002/mc.22714 28796401 PMC5716859

[B71] Keum Y. S. (2011). Regulation of the Keap1/Nrf2 system by chemopreventive sulforaphane: implications of posttranslational modifications. Ann. N. Y. Acad. Sci. 1229, 184–189. 10.1111/j.1749-6632.2011.06092.x 21793854

[B72] Konić-Ristić A. Stanojković T. Srdić-Rajić T. Dilber S. Đorđević B. Stanković I. (2016). *In* vitro assessment of antiproliferative action selectivity of dietary isothiocyanates for tumor versus normal human cells. Vojnosanit. Pregl. 73, 636–642. 10.2298/VSP141103066K 29314795

[B73] Krishnan M. Saraswathy S. Singh S. Saggu G. K. Kalra N. Agrawala P. K. (2024). Sulforaphane inhibits histone deacetylase causing cell cycle arrest and apoptosis in oral squamous carcinoma cells. Med. J. Armed Forces India 80, 412–419. 10.1016/j.mjafi.2022.03.005 39071758 PMC11280278

[B74] Kumari A. Bhawal S. Kapila S. Yadav H. Kapila R. (2022). Health-promoting role of dietary bioactive compounds through epigenetic modulations: a novel prophylactic and therapeutic approach. Crit. Rev. Food Sci. Nutr. 62, 619–639. 10.1080/10408398.2020.1825286 33081489

[B75] Labudda M. Rybarczyk-Płońska A. Sobieszek K. A. Niedziński T. Wurlitzer W. B. Muszyńska E. (2026). The chemopreventive and anticancer potential of glucosinolates and their hydrolysis products from cruciferous vegetables. Nutrients 18, 751. 10.3390/nu18050751 41829922 PMC12987301

[B76] Lampe J. W. (2009). Interindividual differences in response to plant-based diets: implications for cancer risk. Am. J. Clin. Nutr. 89, 1553s–1557s. 10.3945/ajcn.2009.26736D 19297461 PMC2677005

[B77] Lattouf H. Poulard C. Le Romancer M. (2019). PRMT5 prognostic value in cancer. Oncotarget 10, 3151–3153. 10.18632/oncotarget.26883 31139329 PMC6516714

[B78] Lee E. Yang D. Hong J. H. (2025a). Prominent naturally derived oxidative-stress-targeting drugs and their applications in cancer treatment. Antioxidants (Basel) 14, 49. 10.3390/antiox14010049 39857383 PMC11760868

[B79] Lee J. Kim J. Hwang I. (2025b). Targeting PRMT5 in cancer: mechanistic insights and clinical progress. Biomed. Pharmacother. 193, 118754. 10.1016/j.biopha.2025.118754 41223761

[B80] Lenzi M. Fimognari C. Hrelia P. (2014). Sulforaphane as a promising molecule for fighting cancer. Cancer Treat. Res. 159, 207–223. 10.1007/978-3-642-38007-5_12 24114482

[B81] Li W. Jain M. R. Chen C. Yue X. Hebbar V. Zhou R. (2005). Nrf2 possesses a redox-insensitive nuclear export signal overlapping with the leucine zipper motif. J. Biol. Chem. 280, 28430–28438. 10.1074/jbc.M410601200 15917227

[B82] Li Y. Chen X. Lu C. (2021). The interplay between DNA and histone methylation: molecular mechanisms and disease implications. EMBO Rep. 22, e51803. 10.15252/embr.202051803 33844406 PMC8097341

[B83] Li S. Khoi P. N. Yin H. Sah D. K. Kim N. H. Lian S. (2022). Sulforaphane suppresses the nicotine-induced expression of the matrix Metalloproteinase-9 via inhibiting ROS-mediated AP-1 and NF-κB signaling in human gastric cancer cells. Int. J. Mol. Sci. 23, 5172. 10.3390/ijms23095172 35563563 PMC9099819

[B84] Li J. Liu J. Wang Z. Zhao M. You M. Fu Z. (2026). Sulforaphane synergizes with PD-1 blockade through activating CD8(+) T cells in non-small cell lung cancer: preclinical and clinical investigations. MedComm (2020) 7, e70688. 10.1002/mco2.70688 41930330 PMC13042748

[B85] Lin J. Zhang H. Kang H. Chen Y. (2026). Current status and prospects of methylthioadenosine phosphorylase/protein arginine methyltransferase 5 target therapy. Biochem. Pharmacol. 244, 117561. 10.1016/j.bcp.2025.117561 41314432

[B86] Liu B. Zhang G. Cui S. Du G. (2022). Upregulation of KIF11 in TP53 mutant glioma promotes tumor stemness and drug resistance. Cell Mol. Neurobiol. 42, 1477–1485. 10.1007/s10571-020-01038-3 33491154 PMC11421707

[B87] Liu P. Zhang B. Li Y. Yuan Q. (2024). Potential mechanisms of cancer prevention and treatment by sulforaphane, a natural small molecule compound of plant-derived. Mol. Med. 30, 94. 10.1186/s10020-024-00842-7 38902597 PMC11191161

[B88] Long J. Liao X. Tang Z. Han K. Chen J. Wang X. (2025). Investigating the clinical efficacy, safety and molecular mechanism of sulforaphane in autism spectrum disorder: an integrated study combining meta-analysis, network pharmacology, and computational biology. BMC Pharmacol. Toxicol. 26, 217. 10.1186/s40360-025-01052-5 41275316 PMC12751817

[B89] Lu W. Cui J. Wang W. Hu Q. Xue Y. Liu X. (2024a). PPIA dictates NRF2 stability to promote lung cancer progression. Nat. Commun. 15, 4703. 10.1038/s41467-024-48364-4 38830868 PMC11148020

[B90] Lu X. Zhang C. Zhu L. Wang S. Zeng L. Zhong W. (2024b). TBL2 promotes tumorigenesis via PRMT5/WDR77-Mediated AKT activation in breast cancer. Adv. Sci. (Weinh) 11, e2400160. 10.1002/advs.202400160 39499734 PMC11653647

[B91] Lubelska K. Wiktorska K. Mielczarek L. Milczarek M. Zbroińska-Bregisz I. Chilmonczyk Z. (2016). Sulforaphane regulates NFE2L2/Nrf2-Dependent xenobiotic metabolism phase II and phase III enzymes differently in human colorectal cancer and untransformed epithelial Colon cells. Nutr. Cancer 68, 1338–1348. 10.1080/01635581.2016.1224369 27636860

[B92] Lv Y. He J. Peng J. Li H. Yang Y. Liang Z. (2025). Gut microbiota-related glutathione metabolism is key mechanism for sulforaphane ameliorating ulcerative colitis. J. Nutr. Biochem. 146, 110042. 10.1016/j.jnutbio.2025.110042 40714240

[B93] MacArthur I. C. Dawlaty M. M. (2021). TET enzymes and 5-Hydroxymethylcytosine in neural progenitor cell biology and neurodevelopment. Front. Cell Dev. Biol. 9, 645335. 10.3389/fcell.2021.645335 33681230 PMC7930563

[B94] Meeran S. M. Patel S. N. Tollefsbol T. O. (2010). Sulforaphane causes epigenetic repression of hTERT expression in human breast cancer cell lines. PLoS One 5, e11457. 10.1371/journal.pone.0011457 20625516 PMC2897894

[B95] Melchini A. Costa C. Traka M. Miceli N. Mithen R. De Pasquale R. (2009). Erucin, a new promising cancer chemopreventive agent from rocket salads, shows anti-proliferative activity on human lung carcinoma A549 cells. Food Chem. Toxicol. 47, 1430–1436. 10.1016/j.fct.2009.03.024 19328833

[B96] Melchini A. Needs P. W. Mithen R. F. Traka M. H. (2012). Enhanced in vitro biological activity of synthetic 2-(2-pyridyl) ethyl isothiocyanate compared to natural 4-(methylsulfinyl) butyl isothiocyanate. J. Med. Chem. 55, 9682–9692. 10.1021/jm300929v 22998472

[B97] Mishra S. Verma S. S. Rai V. Awasthee N. Chava S. Hui K. M. (2019). Long non-coding RNAs are emerging targets of phytochemicals for cancer and other chronic diseases. Cell Mol. Life Sci. 76, 1947–1966. 10.1007/s00018-019-03053-0 30879091 PMC7775409

[B98] Mitsiogianni M. Trafalis D. T. Franco R. Zoumpourlis V. Pappa A. Panayiotidis M. I. (2021). Sulforaphane and iberin are potent epigenetic modulators of histone acetylation and methylation in malignant melanoma. Eur. J. Nutr. 60, 147–158. 10.1007/s00394-020-02227-y 32215717

[B99] Mordecai J. Ullah S. Ahmad I. (2023). Sulforaphane and its protective role in prostate cancer: a mechanistic approach. Int. J. Mol. Sci. 24, 6979. 10.3390/ijms24086979 37108142 PMC10138336

[B100] Mulvaney K. M. Blomquist C. Acharya N. Li R. Ranaghan M. J. O'Keefe M. (2021). Molecular basis for substrate recruitment to the PRMT5 methylosome. Mol. Cell 81, 3481–3495.e7. 10.1016/j.molcel.2021.07.019 34358446 PMC9016627

[B101] Mutlu M. Raza U. Saatci Ö. Eyüpoğlu E. Yurdusev E. Şahin Ö. (2016). miR-200c: a versatile watchdog in cancer progression, EMT, and drug resistance. J. Mol. Med. Berl. 94, 629–644. 10.1007/s00109-016-1420-5 27094812

[B102] Myzak M. C. Dashwood W. M. Orner G. A. Ho E. Dashwood R. H. (2006). Sulforaphane inhibits histone deacetylase in vivo and suppresses tumorigenesis in Apc-minus mice. Faseb J. 20, 506–508. 10.1096/fj.05-4785fje 16407454 PMC2373266

[B103] Niture S. K. Velu C. S. Smith Q. R. Bhat G. J. Srivenugopal K. S. (2007). Increased expression of the MGMT repair protein mediated by cysteine prodrugs and chemopreventative natural products in human lymphocytes and tumor cell lines. Carcinogenesis 28, 378–389. 10.1093/carcin/bgl155 16950796

[B104] Okonkwo A. Mitra J. Johnson G. S. Li L. Dashwood W. M. Hegde M. L. (2018). Heterocyclic analogs of sulforaphane trigger DNA damage and impede DNA repair in Colon cancer cells: interplay of HATs and HDACs. Mol. Nutr. Food Res. 62, e1800228. 10.1002/mnfr.201800228 29924908 PMC6553464

[B105] Otoo R. A. Allen A. R. (2023). Sulforaphane's multifaceted potential: from neuroprotection to anticancer action. Molecules 28, 6902. 10.3390/molecules28196902 37836745 PMC10574530

[B106] Recillas-Targa F. (2022). Cancer epigenetics: an overview. Arch. Med. Res. 53, 732–740. 10.1016/j.arcmed.2022.11.003 36411173

[B107] Rodon J. Johnson M. L. George B. Shah P. A. Arbour K. C. (2026). MTAP deletion in oncogenesis: a synthetic lethality scenario. Cancer Res. 86, 1558–1569. 10.1158/0008-5472.can-25-2126 41512197 PMC13044523

[B108] Royce S. G. Licciardi P. V. Beh R. C. Bourke J. E. Donovan C. Hung A. (2022). Sulforaphane prevents and reverses allergic airways disease in mice via anti-inflammatory, antioxidant, and epigenetic mechanisms. Cell Mol. Life Sci. 79, 579. 10.1007/s00018-022-04609-3 36319916 PMC11803010

[B109] Sabnis R. W. (2021). Novel PRMT5 inhibitors for treating cancer. ACS Med. Chem. Lett. 12, 1537–1538. 10.1021/acsmedchemlett.1c00512 34676035 PMC8521605

[B110] Saha K. Fisher M. L. Adhikary G. Grun D. Eckert R. L. (2017). Sulforaphane suppresses PRMT5/MEP50 function in epidermal squamous cell carcinoma leading to reduced tumor formation. Carcinogenesis 38, 827–836. 10.1093/carcin/bgx044 28854561 PMC5862259

[B111] Saito A. Ishikawa S. Yang K. Sawa A. Ishizuka K. (2025). Sulforaphane as a potential therapeutic agent: a comprehensive analysis of clinical trials and mechanistic insights. J. Nutr. Sci. 14, e65. 10.1017/jns.2025.10033 40988712 PMC12451241

[B112] Sato K. Kihara H. Kumazawa Y. Tatara K. (2021). Oral chronic sulforaphane effects on heavy resistance exercise: implications for inflammatory and muscle damage parameters in young practitioners. Nutrition 90, 111266. 10.1016/j.nut.2021.111266 34004418

[B113] Schaer D. J. Schulthess-Lutz N. Baselgia L. Kunasingam K. Humar R. Hansen K. (2025). Stress-NRF2 response axis polarizes tumor macrophages and undermines immunotherapy. J. Immunother. Cancer 13, e013063. 10.1136/jitc-2025-013063 41176316 PMC12581087

[B114] Schneider C. Spielmann V. Braun C. J. Schneider G. (2025). PRMT5 inhibitors: therapeutic potential in pancreatic cancer. Transl. Oncol. 55, 102366. 10.1016/j.tranon.2025.102366 40157258 PMC11995137

[B115] Shah P. A. Rodon J. (2025). Targeting the epigenome and immune evasion: a new chapter in PRMT5 inhibition. Ann. Oncol. 36, 1431–1432. 10.1016/j.annonc.2025.09.014 41188151

[B116] Shahabipour F. Caraglia M. Majeed M. Derosa G. Maffioli P. Sahebkar A. (2017). Naturally occurring anti-cancer agents targeting EZH2. Cancer Lett. 400, 325–335. 10.1016/j.canlet.2017.03.020 28323035

[B117] Shen G. Khor T. O. Hu R. Yu S. Nair S. Ho C. T. (2007). Chemoprevention of familial adenomatous polyposis by natural dietary compounds sulforaphane and dibenzoylmethane alone and in combination in ApcMin/+ mouse. Cancer Res. 67, 9937–9944. 10.1158/0008-5472.CAN-07-1112 17942926

[B118] Smith C. R. Aranda R. Bobinski T. P. Briere D. M. Burns A. C. Christensen J. G. (2022). Fragment-based discovery of MRTX1719, a synthetic lethal inhibitor of the PRMT5•MTA complex for the treatment of MTAP-deleted cancers. J. Med. Chem. 65, 1749–1766. 10.1021/acs.jmedchem.1c01900 35041419

[B119] Son Y. Hong S. Jang W. Kim S. Lee H. Lee S. (2026). Global, regional, and national burden of cervical cancer in 2022 and projections to 2050: a population-based analysis of GLOBOCAN. Int. J. Gynecol. Cancer 36, 102751. 10.1016/j.ijgc.2025.102751 41259846

[B120] Song P. Yang F. (2026). Protein arginine methyltransferase 5 as a novel therapeutic target in solid tumors. Genes Dis. 13, 101796. 10.1016/j.gendis.2025.101796 41078957 PMC12513006

[B121] Stopa N. Krebs J. E. Shechter D. (2015). The PRMT5 arginine methyltransferase: many roles in development, cancer and beyond. Cell Mol. Life Sci. 72, 2041–2059. 10.1007/s00018-015-1847-9 25662273 PMC4430368

[B122] Su X. Jiang X. Meng L. Dong X. Shen Y. Xin Y. (2018). Anticancer activity of sulforaphane: the epigenetic mechanisms and the Nrf2 signaling pathway. Oxid. Med. Cell Longev. 2018, 5438179. 10.1155/2018/5438179 29977456 PMC6011061

[B123] Su Z. Tian M. Shibata E. Shibata Y. Yang T. Wang Z. (2025). Regulation of epigenetics and chromosome structure by human ORC2. Cell Rep. 44, 115816. 10.1016/j.celrep.2025.115816 40504688 PMC12360442

[B124] Surh Y. J. (2003). Cancer chemoprevention with dietary phytochemicals. Nat. Rev. Cancer 3, 768–780. 10.1038/nrc1189 14570043

[B125] Thakkar A. Chenreddy S. Wang J. Prabhu S. (2015). Evaluation of ibuprofen loaded solid lipid nanoparticles and its combination regimens for pancreatic cancer chemoprevention. Int. J. Oncol. 46, 1827–1834. 10.3892/ijo.2015.2879 25652350

[B126] Thaler R. Maurizi A. Roschger P. Sturmlechner I. Khani F. Spitzer S. (2016). Anabolic and antiresorptive modulation of bone homeostasis by the epigenetic modulator sulforaphane, a naturally occurring isothiocyanate. J. Biol. Chem. 291, 6754–6771. 10.1074/jbc.M115.678235 26757819 PMC4807263

[B127] Tortorella S. M. Royce S. G. Licciardi P. V. Karagiannis T. C. (2015). Dietary sulforaphane in cancer chemoprevention: the role of epigenetic regulation and HDAC inhibition. Antioxid. Redox Signal 22, 1382–1424. 10.1089/ars.2014.6097 25364882 PMC4432495

[B128] Tsai J. Y. Tsai S. H. Wu C. C. (2019). The chemopreventive isothiocyanate sulforaphane reduces anoikis resistance and anchorage-independent growth in non-small cell human lung cancer cells. Toxicol. Appl. Pharmacol. 362, 116–124. 10.1016/j.taap.2018.10.020 30365975

[B129] Turrini E. Ferruzzi L. Fimognari C. (2014). Natural compounds to overcome cancer chemoresistance: toxicological and clinical issues. Expert Opin. Drug Metab. Toxicol. 10, 1677–1690. 10.1517/17425255.2014.972933 25339439

[B130] Wagner K. D. Wagner N. (2022). The senescence markers p16INK4A, p14ARF/p19ARF, and p21 in organ development and homeostasis. Cells 11, 1966. 10.3390/cells11121966 35741095 PMC9221567

[B131] Wang X. J. Sun Z. Chen W. Li Y. Villeneuve N. F. Zhang D. D. (2008). Activation of Nrf2 by arsenite and monomethylarsonous acid is independent of Keap1-C151: enhanced Keap1-Cul3 interaction. Toxicol. Appl. Pharmacol. 230, 383–389. 10.1016/j.taap.2008.03.003 18417180 PMC2610481

[B132] Wang H. Khor T. O. Yang Q. Huang Y. Wu T. Y. Saw C. L. (2012). Pharmacokinetics and pharmacodynamics of phase II drug metabolizing/antioxidant enzymes gene response by anticancer agent sulforaphane in rat lymphocytes. Mol. Pharm. 9, 2819–2827. 10.1021/mp300130k 22931102 PMC3580178

[B133] Wang D. X. Zou Y. J. Zhuang X. B. Chen S. X. Lin Y. Li W. L. (2017). Sulforaphane suppresses EMT and metastasis in human lung cancer through miR-616-5p-mediated GSK3β/β-catenin signaling pathways. Acta Pharmacol. Sin. 38, 241–251. 10.1038/aps.2016.122 27890917 PMC5309754

[B134] Wang J. Tian K. Y. Fang Y. Chang H. M. Han Y. N. Chen F. Q. (2021). Sulforaphane attenuates cisplatin-induced hearing loss by inhibiting histone deacetylase expression. Int. J. Immunopathol. Pharmacol. 35, 20587384211034086. 10.1177/20587384211034086 34344210 PMC8351026

[B135] Wang Q. Li D. Liu L. Shan Y. Bao Y. (2024). Dietary isothiocyanates and anticancer agents: exploring synergism for improved cancer management. Front. Nutr. 11, 1386083. 10.3389/fnut.2024.1386083 38919393 PMC11196812

[B136] Waqas F. H. Silva da Costa L. Zapatero-Belinchón F. J. Carter-Timofte M. E. Lasswitz L. van der Horst D. (2026). NRF2 activators and the inhibitor of nuclear export, selinexor, restrict coronaviruses by targeting a network involving ACE2, TMPRSS2, and XPO1 through an NRF2-independent mechanism. Commun. Biol. 9, 384. 10.1038/s42003-026-09724-6 41721027 PMC12993055

[B137] Wu Q. Schapira M. Arrowsmith C. H. Barsyte-Lovejoy D. (2021). Protein arginine methylation: from enigmatic functions to therapeutic targeting. Nat. Rev. Drug Discov. 20, 509–530. 10.1038/s41573-021-00159-8 33742187

[B138] Xu Y. Han X. Li Y. Min H. Zhao X. Zhang Y. (2019). Sulforaphane mediates glutathione depletion via polymeric nanoparticles to restore cisplatin chemosensitivity. ACS Nano. 13, 13445–13455. 10.1021/acsnano.9b07032 31670945

[B139] Yang L. Ma D. W. Cao Y. P. Li D. Z. Zhou X. Feng J. F. (2021). PRMT5 functionally associates with EZH2 to promote colorectal cancer progression through epigenetically repressing CDKN2B expression. Theranostics. 11, 3742–3759. 10.7150/thno.53023 33664859 PMC7914347

[B140] Yuan J. M. Kensler T. W. Dacic S. Hartman D. J. Wang R. Balogh P. A. (2025). Randomized phase II clinical trial of sulforaphane in former smokers at high risk for lung cancer. Cancer Prev. Res. (Phila) 18, 335–345. 10.1158/1940-6207.capr-24-0386 40041932 PMC13321263

[B141] Zambrano V. Bustos R. Mahn A. (2019). Insights about stabilization of sulforaphane through microencapsulation. Heliyon 5, e02951. 10.1016/j.heliyon.2019.e02951 31844781 PMC6895643

[B142] Zambrano V. Bustos R. Arozarena Y. Mahn A. (2023). Optimization of a microencapsulation process using oil-in-water (O/W) emulsion to increase thermal stability of sulforaphane. Foods 12, 3869. 10.3390/foods12203869 37893763 PMC10606704

[B143] Zamora-Fuentes J. M. Hernández-Lemus E. Espinal-Enríquez J. (2023). Methylation-related genes involved in renal carcinoma progression. Front. Genet. 14, 1225158. 10.3389/fgene.2023.1225158 37693315 PMC10486271

[B144] Zanichelli F. Capasso S. Cipollaro M. Pagnotta E. Cartenì M. Casale F. (2012). Dose-dependent effects of R-sulforaphane isothiocyanate on the biology of human mesenchymal stem cells, at dietary amounts, it promotes cell proliferation and reduces senescence and apoptosis, while at anti-cancer drug doses, it has a cytotoxic effect. Age (Dordr) 34, 281–293. 10.1007/s11357-011-9231-7 21465338 PMC3312628

[B145] Zeng H. Long Y. Peng Y. Chen Y. (2026). Update on the development of targeting arginine methyltransferase PRMT5 in cancer research. Curr. Top. Med. Chem. 10.2174/0115680266473173260408073728 42163734

[B146] Zhang D. D. Hannink M. (2003). Distinct cysteine residues in Keap1 are required for Keap1-dependent ubiquitination of Nrf2 and for stabilization of Nrf2 by chemopreventive agents and oxidative stress. Mol. Cell Biol. 23, 8137–8151. 10.1128/mcb.23.22.8137-8151.2003 14585973 PMC262403

[B147] Zhang Y. Tang L. Gonzalez V. (2003). Selected isothiocyanates rapidly induce growth inhibition of cancer cells. Mol. Cancer Ther. 2, 1045–1052. 14578469

[B148] Zhang Z. Atwell L. L. Farris P. E. Ho E. Shannon J. (2016). Associations between cruciferous vegetable intake and selected biomarkers among women scheduled for breast biopsies. Public Health Nutr. 19, 1288–1295. 10.1017/S136898001500244X 26329135 PMC10271091

[B149] Zhang Y. Ai P. Chen S. Z. Lei S. Y. (2023). Sulforaphane suppresses skin squamous cell carcinoma cells proliferation through miR-199a-5p/Sirt1/CD44ICD signaling pathway. Immunopharmacol. Immunotoxicol. 45, 52–60. 10.1080/08923973.2022.2112221 35947042

[B150] Zhao Q. Rank G. Tan Y. T. Li H. Moritz R. L. Simpson R. J. (2009). PRMT5-mediated methylation of histone H4R3 recruits DNMT3A, coupling histone and DNA methylation in gene silencing. Nat. Struct. Mol. Biol. 16, 304–311. 10.1038/nsmb.1568 19234465 PMC5120857

[B151] Zhao Z. Chen Q. Qiao X. Wang J. Ali Z. T. A. Li J. (2025). Sulforaphane in cancer precision medicine: from biosynthetic origins to multiscale mechanisms and clinical translation. Front. Immunol. 16, 1702860. 10.3389/fimmu.2025.1702860 41246347 PMC12611815

[B152] Zhou J. W. Wang M. Sun N. X. Qing Y. Yin T. F. Li C. (2019). Sulforaphane-induced epigenetic regulation of Nrf2 expression by DNA methyltransferase in human Caco-2 cells. Oncol. Lett. 18, 2639–2647. 10.3892/ol.2019.10569 31452747 PMC6676568

